# Filling reference gaps via assembling DNA barcodes using high-throughput sequencing—moving toward barcoding the world

**DOI:** 10.1093/gigascience/gix104

**Published:** 2017-10-25

**Authors:** Shanlin Liu, Chentao Yang, Chengran Zhou, Xin Zhou

**Affiliations:** Beijing Advanced Innovation Center for Food Nutrition and Human Health, College of Plant Protection, China Agricultural University, Beijing 100193, People's Republic of China; BGI-Shenzhen, Shenzhen, 518083, China; National Engineering Research Center for Fruit and Vegetable Processing, China Agricultural University, Beijing 100193, People's Republic of China; Centre for GeoGenetics, Natural History Museum of Denmark, University of Copenhagen, 1350, Copenhagen, Denmark; Key Laboratory of Bio-Resources and Eco-Environment, Ministry of Education, College of Life Sciences, Sichuan University, Chengdu 610065, China

**Keywords:** DNA Barcode, High-throughput sequencing, COI, Biodiveristy, meta-barcoding

## Abstract

Over the past decade, biodiversity researchers have dedicated tremendous efforts to constructing DNA reference barcodes for rapid species registration and identification. Although analytical cost for standard DNA barcoding has been significantly reduced since early 2000, further dramatic reduction in barcoding costs is unlikely because Sanger sequencing is approaching its limits in throughput and chemistry cost. Constraints in barcoding cost not only led to unbalanced barcoding efforts around the globe, but also prevented high-throughput sequencing (HTS)–based taxonomic identification from applying binomial species names, which provide crucial linkages to biological knowledge. We developed an Illumina-based pipeline, HIFI-Barcode, to produce full-length Cytochrome c oxidase subunit I (COI) barcodes from pooled polymerase chain reaction amplicons generated by individual specimens. The new pipeline generated accurate barcode sequences that were comparable to Sanger standards, even for different haplotypes of the same species that were only a few nucleotides different from each other. Additionally, the new pipeline was much more sensitive in recovering amplicons at low quantity. The HIFI-Barcode pipeline successfully recovered barcodes from more than 78% of the polymerase chain reactions that didn’t show clear bands on the electrophoresis gel. Moreover, sequencing results based on the single molecular sequencing platform Pacbio confirmed the accuracy of the HIFI-Barcode results. Altogether, the new pipeline can provide an improved solution to produce full-length reference barcodes at about one-tenth of the current cost, enabling construction of comprehensive barcode libraries for local fauna, leading to a feasible direction for DNA barcoding global biomes.

## Background

Over the past decade, biodiversity research has seen paradigm shifts in methodology developments and applications [1], where standard DNA sequences, e.g., DNA barcodes, are adopted for fast and accurate taxonomic diagnoses and high-throughput sequencing (HTS) platforms are employed in analysis of complex biological samples, including bulk samples [2, 3], environmental DNA (eDNA [4]), invertebrate-derived DNA (iDNA [5, 6]), etc. DNA barcode reference libraries have been constructed globally via a synergistic effort, resulting in well-curated, centralized barcode registration databases, e.g., the Barcode of Life Data systems [7], which has recently reached a milestone for 5 million barcodes, covering ca. 0.26 million species (accessed in July 2017). These DNA barcodes have been effectively facilitating species identification, phylogenetic reconstruction [8], and understanding of interspecific interactions and community structures [1].

Along with the rapid accumulation of global barcode references for various taxon groups, significant effort has been made in digitalizing biomes, e.g., sequencing all taxa of particular lineages found in entire ranges of national parks or islands [9]. Early efforts in barcoding biomes have employed standard Sanger sequencing-based approaches to characterizing focal fauna [10–12]. Alternatively, boosted by HTS technologies, DNA metabarcoding and mitochondrial metagenomics (mitochondrial genome skimming) have been applied in investigations of local biodiversity and in evaluation of biological managements [13–17]. These practices allow investigators to rapidly understand species richness or even approximation for species evenness and/or biomass for complex biological samples [4, 18]. A typical dilemma, however, is the lack of local barcode references from which HTS biodiversity analysis could draw conclusions on species occurrences. This is primarily due to unbalanced barcoding efforts around the globe, where regions in desperate need for biodiversity research are typically suffering from insufficient funding for taxonomy work, especially for DNA-based studies. Consequently, HTS-based taxonomic registrations are often constrained to applying molecular operational units (OTUs) instead of binomial species names, and are therefore unable to associate existing biological and ecological knowledge to the resultant diversity composition.

Admittedly, the analytical cost for standard DNA barcoding has been significantly reduced since early 2000, a result of the development of centralized and industrialized barcoding facilities and automated pipelines [1]. Currently, the average production cost for a reference barcode is ca. $10 USD, excluding the costs for sample collection and handling. Further dramatic reductions in barcoding costs are unlikely because Sanger sequencing technology is approaching its limits in throughput and associated chemistry cost. It is estimated that 100 million specimens would need to be sequenced to complete the global barcode registration [1], which translates into a roughly $1 billion budget merely for reference constructions. A similar challenge was seen in the sequencing of the first human genome, where an initial budget of more than $3 billion USD was estimated based on the application of Sanger sequencing [19]. Thanks to the advent of HTS technologies over the past decade, the current cost of a human genome is now within the range of $1000 USD, if not less.

An early study using HTS in generating barcodes from single specimens employed the Roche 454 platform [20], which was rapidly phased out due to limited throughput capacity (hence high chemistry cost). Illumina platforms (e.g., Hiseq and Miseq) have been primarily applied in recent practice [21], but these are constrained by relatively short read lengths (100–300 bps). Even with the most recent Miseq model at 300 bp paired-end (PE) sequencing, full-length barcodes (e.g., ∼700 bps for Cytochrome c oxidase subunit I (COI) including primers) are beyond the sequencing range. Therefore, existing pipelines are forced to produce a fragment of the standard barcodes (e.g., 313 bp [22]) or to apply 2 rounds of polymerase chain reaction (PCR) amplifications, each targeting a proportion of the full barcodes [21]. Obviously, full-length barcodes are desired for constructing barcode references, and extra amplification procedures should be avoided when possible for cost control and simplification of pipelines. In particular, efficient primers might be difficult to identify in the mid-COI barcode region across taxon groups. Alternatively, short HTS reads can be assembled into much longer scaffolds, which is a standard practice in *de novo* genome or transcriptome assembling. In fact, a specific assembly algorithm, SOAPBarcode, has been developed for recovering full-length barcodes from pooled arthropod samples [23].

**Figure 1:**
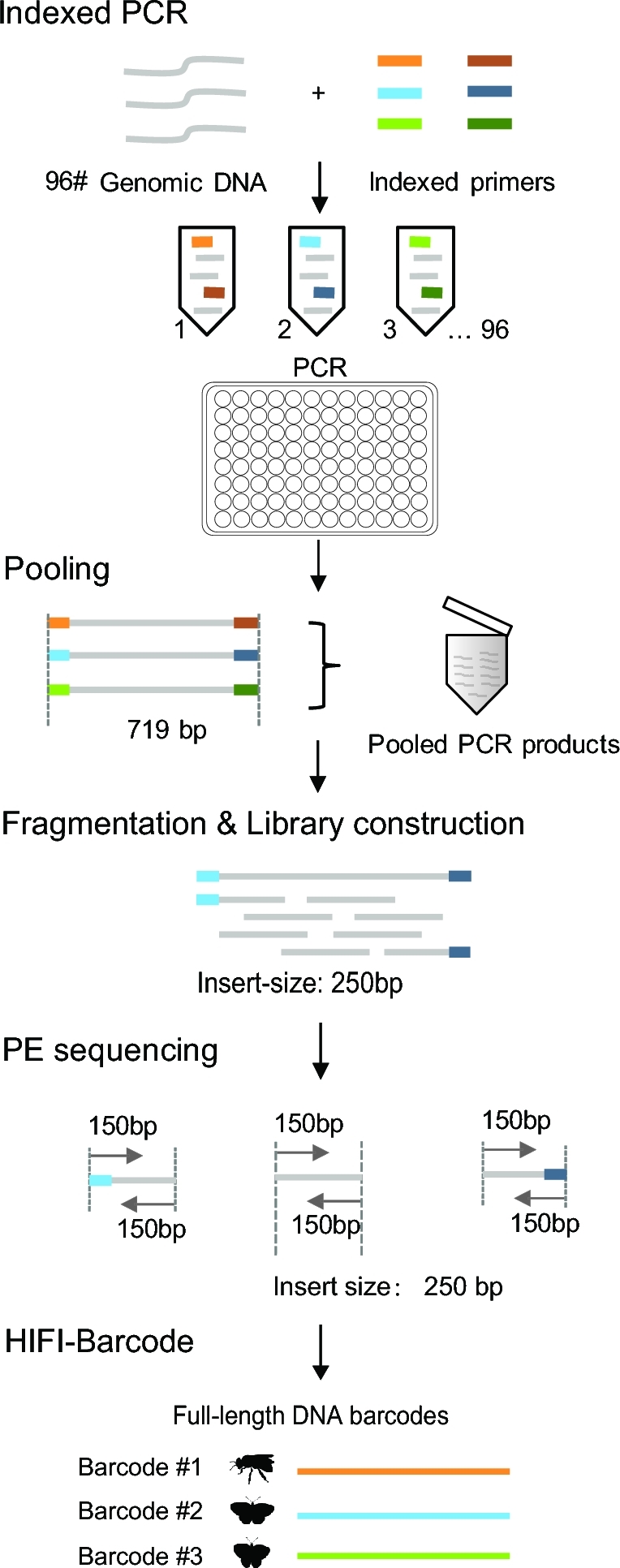
Schematic illustration of the HIFI-Barcode pipeline.

Here, we introduce a more straightforward and cost-efficient HTS pipeline that generates full-length reference barcodes—HIFI-Barcode (Fig. 1). Briefly, individual genomic DNA was extracted separately and amplified on a 96-well plate using 96 sets of uniquely tagged primers. Amplicons were then pooled and sequenced on an Illumina Hiseq 4000 platform at 150 PE. Mixed HTS reads were assembled using a customized bioinformatics pipeline to obtain the barcode sequence for each individual. Compared with the aforementioned studies [21, 22], our method can deliver standard full-length barcodes via a single PCR reaction, and the sequencing is carried out on the HiSeq platform, the most cost-effective HTS platform currently available. Using Sanger barcodes as the gold standard, the new pipeline can generate accurate individual barcode sequences, even for haplotypes of the same species that are only a few nucleotides different from each other. Additionally, the new pipeline is much more sensitive than Sanger in recovering amplicons at low quantity. More than 78% (25/32) of the “failed” PCR amplicons (those without clear bands on an electrophoresis gel) were successfully recovered at high quality using the new pipeline. In addition, the single-molecule sequencing platform Pacbio has also been adopted in our study to evaluate the accuracy of the HIFI-Barcode method. Altogether, the new pipeline can provide an alternative solution to producing full-length reference barcodes at about one-tenth of the current cost, enabling larger-scale biodiversity barcoding initiatives, especially for areas where DNA references are scarce.

## Materials and Methods

### DNA preparation

Insect specimens were collected at Laohegou Natural Reserve, Sichuan Province, China. Genomic DNA was extracted in an independent study using the Glass Fiber Plate method following the manufacturer's protocol [24]. Two 96-well plates were prepared for the current work: 1 plate containing 96 high-quality lepidopteran DNA (showing a clear band of standard DNA barcode amplicon on an electrophoresis gel) was used to evaluate the accuracy of our HTS method using Sanger barcodes as the gold standard; a second plate containing 95 randomly selected DNA (mostly dipterans) regardless of quality and PCR yields plus a blank control was prepared to examine the success rate of our HTS method compared with the classic Sanger approach.

### DNA amplification and sequencing

Ninety-six pairs of different tags were added to both ends of a common COI barcode primer set (LCO1490 and HCO2198) (Supplementary Table S1) [25], with each tag containing 5 bps, allowing for ≥2 bp differences from each other. Each PCR reaction contained 1 μL of DNA template, 16.2 μL of molecular biology grade water, 3 μL of ×10 reaction buffer (Mg^2+^ plus), 2.5 μL of dNTPs mix (10 mM), 1 μL of forward and reverse primers (10 mM), and 0.3 μL of TaKaRa Ex Taq polymerase (5 U/μL). The amplification program included a thermocycling profile of 94°C for 1 minute, 5 cycles of 94°C for 30 seconds, 45°C for 40 seconds, and an extension at 72°C for 1 minute, followed by 35 cycles of 94°C for 30 seconds, 51°C for 40 seconds, and 72°C for 1 minute, with a final extension at 72°C for 10 minutes, and finally holding at 12°C. All amplicons were visualized on a 1.2% 96 Agarose E-gel (Biowest Agarose). All PCR products from each plate were pooled using 1 μL per sample, resulting in two 96-μL mixtures, which were sent to BGI and sequenced using a Hiseq 4000. PCR amplicons were fragmented to construct library with an insert size of 250 bp and sequenced with a strategy of 150 PE. A second set of PCR mixtures of the second plate (576 μL, 6 μL per sample) was also sequenced using PacBio RS II at NextOmics.

### HIFI-Barcode assembly

#### Data filtering

Reads of bad-quality were removed from raw data: (i) reads with adapter contamination (≥15-bps alignment length and ≤3 mismatches); (ii) reads with >10 Ns; (iii) reads with >50 bps of low quality (Phred quality score = 2, ASCII 35 “B,” Illumina 1.8+ Phred+33).

#### Read assignment

**Figure 2:**
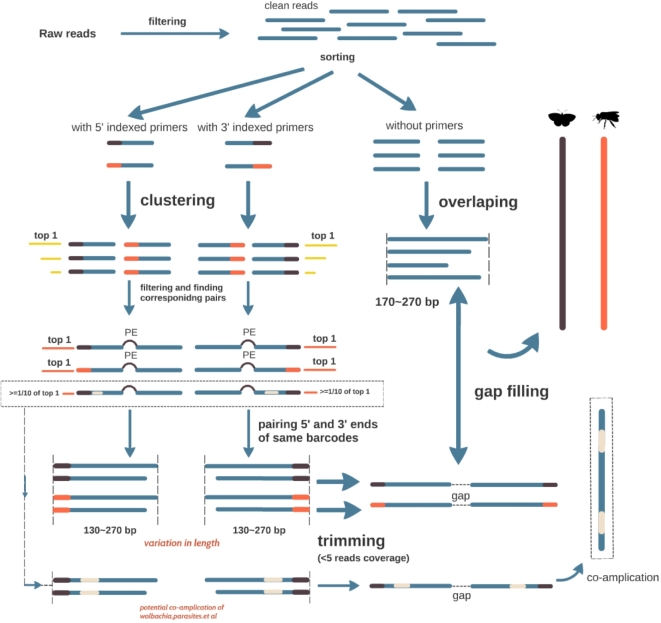
HIFI-Barcode assembly pipeline.

First, reads containing 5΄ and 3΄ ends of each individual were identified based on their unique 5-mer tags and corresponding primer sequences using in-house Perl scripts (see code). Then, for each individual, identical reads were clustered to obtain unique 5΄ and 3΄ sequences. Each individual may contain multiple unique terminal sequences at varied abundances due to haplotype heterogeneity (mitochondrial heteroplasmy) or artefacts (PCR or sequencing errors). Next, the most abundant unique sequence was chosen for the following overlapping and assembly procedures. In addition, if the next most abundant unique sequence had an abundance ≥1/10 of that of the most abundant unique sequence at <98% similarity (sequences were clustered using VSEARCH [26]), it was also retained to confirm identities, e.g., parasites, *Wolbachia*, gut contents that were co-amplified in PCR. After that, corresponding pairs of the previously chosen reads were identified according to their titles, and then paired-end reads were overlapped using COAP [27] with an identity cutoff of 95%. Overlapped reads could vary in sequence length due to insert size fluctuation during ultrasonic shearing. Thus, consensus 5΄ and 3΄ sequences of each individual were achieved using in-house Perl scripts where ends with read coverage <5 were trimmed off (Fig. 2).

#### Gap filling

Algorithm, adopted from SOAPBarcode (Supplementary Fig. S1) [23], was applied to fill the gaps between 5΄ and 3΄ terminal scaffolds of each individual to complete the full-length barcodes. Briefly, for each individual, the 5΄ end was defined as the start point, and the 3΄ end as the end point. Then, the kmer set from de Brujin graph was walked step by step from the start point to the end point to find potential assembly paths. Several strategies were applied to ensure correct paths: (i) kmers of abundance <10% of the average kmer abundance before path bifurcation were removed; (ii) if there was more than 1 out degree remaining after step 1, common reads were counted between different out degrees and the kmer located before the last bifurcation, and the out degrees of common reads <10% of the average abundance were removed; (iii) paths expanding beyond the preset length (standard COI barcode length plus primers) without an end point were removed.

### Data filtering and read assignment for Pacbio

The Pacbio SmrtAnalysis pipeline [28] was adopted to extract 28 770 circular consensus sequences (CCS) from 1.1 G of raw data. Then, CCS of ≥15 passes were chosen for next steps: (i) 22 075 CCS were demultiplexed by their corresponding indices using an in-house Perl script, allowing a maximum of 1 bp deletion at the 5΄ end of forward index or the 3΄ end of reverse index. (ii) For each sample, sequences with a length range out of 658 ± 6 bp were removed, and the remaining unique sequences were sorted by pass numbers and identical sequences were clustered together. (iii) The unique sequence from the most abundant cluster was retained as the correct barcode sequence for each sample.

### Comparisons between HTS, Sanger barcodes, and Pacbio clusters

Barcode sequences obtained by Sanger, the HIFI-Barcode method, and Pacbio were subject to phylogenetic tree constructions using MEGA7 (neighbor-joining and 1000 bootstrap) and iTOL [29]. BWA (BWA, RRID: SCR_010910) [30] was applied to align raw reads to assembled HTS barcodes to examine discrepancies between HTS and Sanger sequences.

The standard operating procedures are also available from the protocols.io repository [31].

## Results

A total of 4 824 443 and 4 439 345 PE reads for the first and second plate were obtained after data filtering, respectively, using Hiseq 4000.

For the first plate, a total of 1 910 616 (39.60%) reads were assigned to their corresponding samples as either a 5΄ or 3΄ end, and 1 898 372 (39.34%) as reads belonging to intermediate regions, while 1 015 455 (21.05%) reads were identified as primer dimers or short PCR chimeras. The abundance of end reads for each sample varies significantly, ranging from 2444 to 64 705. After clustering at 100% similarity for the 5΄ and 3΄ end reads, most samples (61 out of 96) obtained single unique reads after read assignment. The second plate possessed similar read distribution; details of both plates are summarized in Table 1.

**Table 1:** Read distribution of both Illumina and Pacbio platforms

	Raw	Clean	5΄ and	Read	Recovered	Sample	Single	Full-length
	read	read	3΄ read	in-between	indices	size^1^	unique^2^	barcodes
Hiseq 1	8 567 336	4 824 443	1 910 616	1 898 372	96	39 805 (64 705; 2444)	61	96
Hiseq 2	11 531 498	4 439 345	1 306 054	2 676 915	96	27 210 (101 512; 279)	45	88
Pacbio 2*	1 201 158	28 770	26.4	17 102	82	208 (1696; 1)	NA	82
		Total number^3^	Average pass^3^	Assigned^3^				

*Numbers 1 and 2 in this column represent plate ID. (1) Read number possessed by samples formatted as: average (max; min). (2) Number of clusters that left only 1 single representative candidate after read assignment filtering. (3) Statistics of circular consensus sequence.

One cell of Pacbio data containing 28 770 circular CCS from 1 201 158 raw reads was generated for the second plate. CCS reads had an average pass number of 26.5 and were assigned to 82 samples after demultiplexing (Table 1). Note that a single Pacbio sequencing read can reach as long as 40 kb. Therefore, a short CCS read of high quality can be sequenced dozens of times, which in turn effectively corrects sequence errors associated with the platform [32].

### Accuracy and efficiency

**Figure 3:**
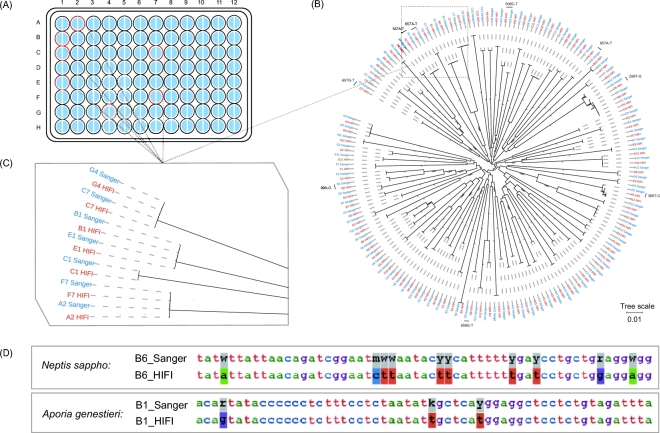
Comparison between HIFI-Barcode and Sanger reference. (A) Success rates of the first plate. For all 96 samples, both Sanger (left semicircle) and HIFI-Barcode (right semicircle) were successful in producing a full-length COI barcode. Samples with red out lining are marked on the phylograms. (B) Phylogenetic tree of all HIFI-Barcodes and Sanger references. (C) Close-up view of representative individuals. (D) Degenerate sites of Sanger references were recuperated by HIFI-Barcodes.

**Figure 4:**
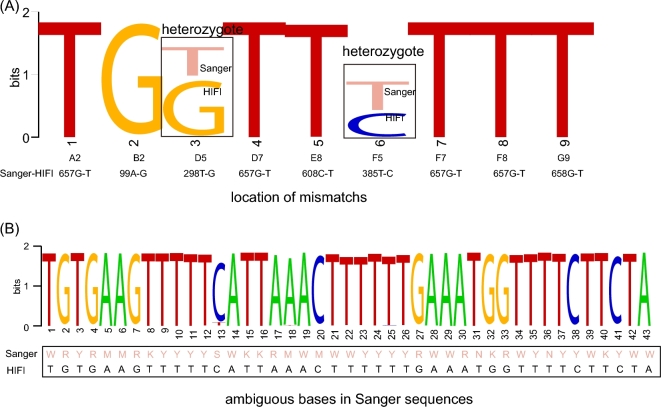
Discrepancies between Sanger and HIFI-Barcodes in the first plate. Entropy weight was calculated based on the strength of read depth by aligning Illumina raw reads onto assembled HIFI-Barcodes, showing potential heteroplasmy (A) and differences between ambiguous Sanger base-calling and specific nucleotide identified in HIFI-Barcodes (B).

Sanger barcodes were obtained from all 96 lepidopteran samples from the first plate (Fig. 3A), including 91 haplotypes and 85 OTUs using a similarity threshold of ≥98%. The HIFI-Barcode assemblies were successful for all 96 samples and showed high accuracy compared with Sanger sequences. Even identical or highly similar barcodes from individuals of the same species were correctly assembled, e.g., A2 vs F7, B1 vs E1, and C7 vs G4 (Fig. 3B and C). A total of 43 ambiguous sites (out of 63 168 bps) found in Sanger barcodes were identified to a specific nucleotide in HIFI-Barcodes (e.g., Figs 3D and 4B). Only 9 HIFI-Barcodes showed a single nucleotide difference from the corresponding Sanger sequences, which could reflect ambiguous base-calling in Sanger sequencing or genuine heteroplasmy in the examined individual. At least 2 of the discrepancies were proven to be heteroplasmy via mapping raw reads against discrepant sites (Fig. 4A).

**Figure 5:**
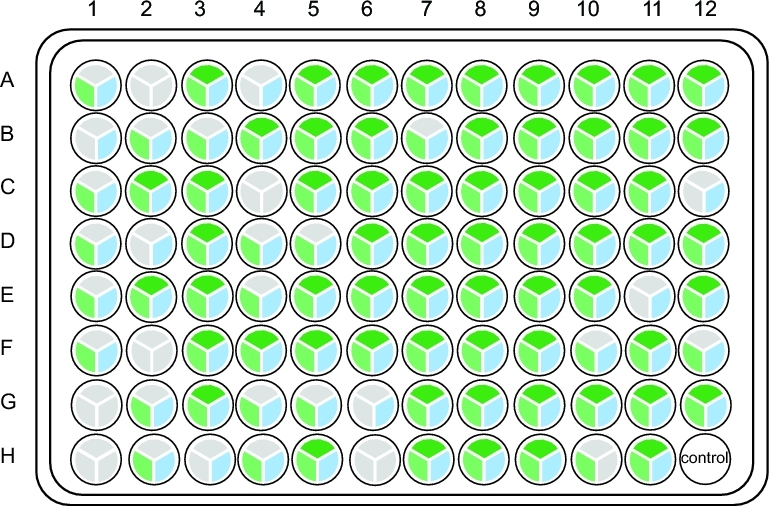
Success rates of the second plate. For each sample, the upper, left, and right pies represent PCR, Pacbio, and HIFI-Barcode, respectively. Gray represents failure, and the others represent success.

In the second plate, samples were randomly selected regardless of their DNA quality and PCR success rates. Sixty-three PCR reactions showed clear bands on the electrophoresis gel (Supplemental Table S2), of which 62 resulted in Sanger barcodes. The HIFI-Barcode pipeline successfully produced full-length HTS sequences for all 62 corresponding Sanger barcodes at high accuracy (56 at 100% match, 5 with 1 mismatch, and 1 with 3 mismatches) (Supplementary Fig. S2). In addition, HIFI-Barcodes were successfully generated from 25 out of the 32 PCR amplicons that had no clear bands (Supplementary Fig. S3, Supplementary Table S2), increasing the overall success rate from 66.32% to 92.63% for the Sanger and HIFI-Barcode methods, respectively (Fig. 5). To further evaluate the accuracy of the newly developed HIFI-Barcode pipeline, especially for those where PCR reactions failed, we also sequenced pooled PCR amplicons using Pacbio. The CCS used in our study had pass numbers >15, which meant the same molecule was sequenced repeatedly, more than 15 times. Thus the consensus nucleotides for each sequence were corrected from sequencing errors associated with the platform (ca. 10% on average). The overall success rate for Pacbio was 86.32%. Of the 25 HIFI-Barcodes where Sanger failed, 18 Pacbio barcodes were obtained. Among these, 10 were identical to the corresponding HIFI-Barcodes; 3 had 1 or 2 sites matched with 1 of the 2 heterozygous alleles from HIFI-Barcodes, and 5 showed errors in amino acid translation (e.g., stop codon), possibly due to sequencing errors in Pacbio (Supplementary Table S3 and Supplementary file S1).

### Nontarget sequences detected by HIFI-Barcode

During the HIFI-Barcode assembly procedure, terminus sequences with ≥1/10 abundance of that of the most abundant scaffolds at <98% similarity were retained for assembly and identity check. This analysis allowed detection of 18 nontarget sequences co-amplified from the 2 plates (Supplemental Table S4), in addition to COI barcodes. Cross-examinations against both NCBI and barcode sequences from the focal plates suggested origins including *Wolbachia* (2), fungus (1), and cross-contamination from adjacent wells (7), as well as potential PCR errors and pseudo-genes (8). The presence of nontarget PCR products from the second plate was further confirmed by Pacbio sequencing at >99% identity, therefore ruling out the likelihood of assembly errors in the HIFI-Barcode pipeline, suggesting that there are co-amplified NUMTs present in PCR products. These low-quantity sequences are likely common in regular PCR-based pipelines and detectable by HTS-based approaches. But they can be easily filtered out from genuine COI barcodes following the pipeline described in this study.

## Discussion

It is widely acknowledged that we have been undergoing unprecedented global biodiversity loss [33]. DNA-based approaches, e.g., DNA barcoding, DNA metabarcoding, mitochondrial metagenomics (mitochondrial genome skimming), have demonstrated efficacy in accelerating biodiversity inventories of large geographical ranges. These standardized and largely automated procedures will provide pivotal information to understand how biodiversity loss is characterized and how to desist from it. New methodologies enable rapid collection of biodiversity and ecology data at a large scale over space and time, which in-turn benefits policy-makers at varied management levels and research groups [34].

Interpreting molecular results using existing knowledge on biology, ecology, and evolution would require a linkage between DNA references and Linnaeus names, which is one of the fundamental roles of DNA barcoding initiatives. The construction of comprehensive barcode references is still, to a large extent, expensive and sometimes prohibitive. This is particularly true for studies targeting a wide range of taxa from a large area of natural habitat. Although the most represented DNA barcode database (BOLD) now hosts barcodes for 0.26 million species, accounting for ca. one-fourth of described species, the chances of encountering a novel barcode are still very high, especially for many biodiversity hotspots. Even if an ecological study focuses on just a small proportion of the focal diversity, it is not uncommon that hundreds to thousands of species would need to be barcoded to draw meaningful conclusions. In addition, multiple individuals of the same species (ideally from distinct populations) would need to be sequenced to reflect intraspecific genetic diversities. There is no consensus on the ideal number of conspecific individuals to be sequenced, but in practice an average of 10 is often followed, while some studies recommend 20 [35], if not a lot more. Therefore, roughly tens of thousands of individuals, requiring hundreds of thousands of dollars (USD), are expected to be sufficient for a regular ecology study, just for the molecular analysis (for a recent example, please see [12]). While the HTS-based approaches have shown promising power in analyzing complex sample mixtures at a much reduced unit cost [2–4, 14], one would still need to establish DNA barcode references to be able to go beyond OTU-based interpretation.

The HIFI-Barcode method, as the results showed, offers a novel route to produce mass volumes of reliable barcode sequences at a significantly reduced cost. The main costs of the HIFI-Barcode pipeline include consumable chemistries, library construction, high-throughput sequencing, and informatics. Despite the increased one-time cost of ordering multiple unique sets of primers, the cost of primers per unit reaction is negligible. Following our protocols, the average cost for a HIFI-Barcode is around $1 USD, as opposed to $10–20 USD using the standard Sanger approach. Further savings on the production cost are achieved by increased success rates, especially for amplicons with low quantity. In our test, ca. one-third of the second plate would have been re-amplified in standard barcoding protocols, using a different set of primers, followed by gel examination, positive picking, PCR purification, and Sanger sequencing.

By complementing the barcode reference library at <1/10 of the current cost, the new approach also reinforces rapid constructions of organelle genomes, e.g., mitochondria and chloroplasts. A number of pilot studies have demonstrated that full mitochondrial genomes can provide elevated power in bulk sample analysis [18, 36]. New approaches to assembling full mito-genomes or the majority of the coding genes have been developed for shotgun sequencing of individual specimens [37], pooled taxa [18, 36], and transcriptomes [37]. In particular, mito-genome assembly through direct shotgun sequencing of mixed taxa can significantly reduce the library construction cost for HTS. Bait sequences, which regularly include standard COI barcodes, are important for assigning mixed mitochondrial scaffolds to a specific taxon. This is critical, especially if the phylogenetic signal of the scaffolds alone is not sufficient to attribute assemblies to species, e.g., when multiple closely related species are pooled. In fact, having multiple bait sequences per species will significantly remove the bioinformatics challenge during the assembly procedure [38], which becomes financially feasible with the help of the HIFI-Barcode pipeline.

Several aspects of our method could be further improved: (i) Multiple barcode markers (e.g., COI, CYTB, 12S, etc.) could be pooled into a single shotgun sequencing effort without increasing tag complexity, which would again alleviate analytical cost. (ii) The pooled PCR amplicons were subject to library construction directly in the present study. The proportion of primer dimers and short PCR chimera reached as high as ca. 21% in our raw reads, which could be easily reduced using size-preference magnetic beads. (iii) The addition of inosine to the 3΄ terminus of the primer could increase its universality and would further elevate the successful rate and efficiency. (iv) Longer tags, allowing for pooling more individuals (e.g., 384-well plate), could further increase the throughput capacity.

In summary, the HIFI-Barcode method provides an HTS-based approach with improved economic efficiency, which allows investigators to produce standard full-length barcodes at ca. one-tenth of the current cost. The new protocol not only generates barcode sequences of high quality that are comparable to Sanger barcodes, but also increases overall sequencing success rates by detecting PCR amplicons in minute quantities. This new method enables construction of comprehensive barcode libraries for local fauna, leading to a feasible direction for DNA barcoding global biomes.

## Availability of source code and requirements

- Project name: HIFI–Barcode project
- Project home pages: https://github.com/comery/HIFI-barcode-hiseq and https://github.com/comery/HIFI-barcode-pacbio
- Operating system(s): Unix, Linux
- Programming language: PERL
- Other requirements: GCC version ≥ 4.4.5
- License: GNU General Public License version 3.0 (GPLv3)
- Any restrictions to use by nonacademics: none

## Availability of Supporting Data

Supporting snapshots of the HIFI-Barcode code and test data are available in the *GigaScience* repository, *Giga*DB [39]. Raw data and sample information are also available from NCBI bioproject PRJNA414137. The standard operating procedure of HIFI-Barcode is also found in the protocols.io protocols repository [31].

## Additional Files

Supplementary Figure S1. Algorithm described in the SOAPBarcode pipeline.

Supplementary Figure S2. Phylogenetic tree of samples sharing Sanger references, HIFI-Barcodes, and Pacbio barcodes.

Supplementary Figure S3. PCR electrophoresis results of the second plate.

Supplementary Table S1. Indexed primer sequences.

Supplementary Table S2. PCR electrophoresis results.

Supplementary Table S3. Comparison of 18 Pacbio barcodes and HIFI-Barcodes.

Supplementary Table S4. Nontarget sequences detected by HIFI-Barcode.

Supplementary File S1. Results of HIFI-Barcode.

## Abbreviations

BOLD: Barcode of Life Data systems; CCSs: circular consensus sequencing; eDNA: environmental DNA; GB: gigabase; HTS: high-throughput sequencing; iDNA: invertebrate-derived DNA; OTUs: molecular operational units; PCR: polymerase chain reaction; PE: paired end.

## Competing interests

The authors declare that they have no competing interests.

## Funding

This work was supported by the China National GeneBank, BGI, and by China Agricultural University through the Chinese Universities Scientific Fund (2017QC114 to X.Z.).

## Author contributions

X.Z. and S.L. designed the study; S.L. coordinated the project and led the analyses; C.Z. and C.Y. led the bench work and contributed to the analyses; S.L., C.Z., and C.Y. formulated the early drafts; and X.Z. revised the manuscript.

## Supplementary Material

gix104_GIGA-D-17-00172_Original-Submission.pdf

gix104_GIGA-D-17-00172_Revision-1.pdf

gix104_GIGA-D-17-00172_Revision-2.pdf

gix104_Response-to-Reviewer-Comments_Original-Submission.pdf

gix104_Response-to-Reviewer-Comments_Revision-1.pdf

gix104_Reviewer-1-Report-(Original-Submission).pdf

gix104_Reviewer-2-Report-(Original-Submission).pdf

Supplementary Table S1. Indexed primer sequences

Supplementary Table S2. PCR electrophoresis results

Supplementary Table S3. Comparison of 18 Pacbio barcodes and HIFI-Barcodes

Supplementary Table S4. Nontarget sequences detected by HIFI-Barcode

Supplementary Figure S1. Algorithm described in the SOAPBarcode pipeline

Supplementary Figure S2. Phylogenetic tree of samples sharing Sanger references, HIFI-Barcodes, and Pacbio barcodes

Supplementary Figure S3. PCR electrophoresis results of the second plate

Supplementary File S1. Results of HIFI-Barcode
