# Supplementary material for: Filling reference gaps via assembling DNA barcodes using high-throughput sequencing—moving toward barcoding the world
Source: Gigascience. 2017 Oct 25;6(12):1–8. doi: 10.1093/gigascience/gix104 (PMC5726475; doi:10.1093/gigascience/gix104)
Supplement: gix104_GIGA-D-17-00172_Original-Submission.pdf [file gix104_giga-d-17-00172_original-submission.pdf]

GigaScience

Filling reference gaps via assembling DNA barcodes using high-throughput sequencing - moving toward barcoding the world

--Manuscript Draft--

Manuscript Number:	GIGA-D-17-00172
Full Title:	Filling reference gaps via assembling DNA barcodes using high-throughput sequencing - moving toward barcoding the world
Article Type:	Research
Funding Information:	the Chinese Universities Scientific Fund (2017QC114) — Not applicable
Abstract:	Over the past decade, biodiversity scientists have dedicated tremendous efforts in constructing DNA reference barcodes for rapid species registration and identification. Although analytical cost for standard DNA barcoding has been significantly reduced since early 2,000, further dramatic reduction on barcoding costs is unlikely because the Sanger sequencing is approaching its limits in throughput and chemistry cost. Constraints in barcoding cost not only led to unbalanced barcoding efforts around the globe, but also refrained High-Throughput-Sequencing (HTS) based taxonomic identification from applying binomial species names, which provide crucial linkages to biological knowledge. We developed an Illumina-based pipeline, HIFI-Barcode, to produce full-length COI barcodes from pooled PCR amplicons generated by individual specimens. The new pipeline generated accurate barcode sequences that were comparable to Sanger standards, even for different haplotypes of the same species that were only a few nucleotides different from each other. Additionally, the new pipeline was much more sensitive in recovering amplicons at low quantity. The HIFI-Barcode pipeline successfully recovered barcodes from over 78% of the PCR reactions that didn't show clear bands on the electrophoresis gel. Moreover, sequencing results based on the single molecular sequencing platform, Pacbio, confirmed the accuracy the HIFI-Barcode results. Altogether, the new pipeline can provide an improved solution to produce full-length reference barcodes at about 1/10 of the current cost, enabling construction of comprehensive barcode libraries for local fauna, leading to a feasible direction for DNA barcoding global biomes.
Corresponding Author:	Xin Zhou<br><br>CHINA
Corresponding Author Secondary Information:	
Corresponding Author's Institution:	
Corresponding Author's Secondary Institution:	
First Author:	Shanlin Liu
First Author Secondary Information:	
Order of Authors:	Shanlin Liu
	Chentao Yang
	Chengran Zhou
	Xin Zhou
Order of Authors Secondary Information:	
Opposed Reviewers:	Mehrdad Hajibabae, Ph.D.<br>Biodiversity Institute of Ontario & Integrative Biology, University of Guelph<br>mhajibab@uoguelph.ca<br>Direct competitor on barcode method development. We request you exclude anyone from this institute from reviewing the manuscript

Powered by Editorial Manager® and ProduXion Manager® from Aries Systems Corporation

Additional Information:	
**Question**	**Response**
Are you submitting this manuscript to a special series or article collection?	No
**Experimental design and statistics**<br><br>Full details of the experimental design and statistical methods used should be given in the Methods section, as detailed in our Minimum Standards Reporting Checklist. Information essential to interpreting the data presented should be made available in the figure legends.<br><br>Have you included all the information requested in your manuscript?	Yes
**Resources**<br><br>A description of all resources used, including antibodies, cell lines, animals and software tools, with enough information to allow them to be uniquely identified, should be included in the Methods section. Authors are strongly encouraged to cite Research Resource Identifiers (RRIDs) for antibodies, model organisms and tools, where possible.<br><br>Have you included the information requested as detailed in our Minimum Standards Reporting Checklist?	Yes
**Availability of data and materials**<br><br>All datasets and code on which the conclusions of the paper rely must be either included in your submission or deposited in publicly available repositories (where available and ethically appropriate), referencing such data using a unique identifier in the references and in the “Availability of Data and Materials” section of your manuscript.<br><br>Have you have met the above requirement as detailed in our Minimum Standards Reporting Checklist?	Yes

Powered by Editorial Manager® and ProduXion Manager® from Aries Systems Corporation

Manuscript

Click here to download Manuscript move closer to barcoding the world 0712.docx

Filling reference gaps via assembling DNA barcodes using high-throughput sequencing – moving toward barcoding the world

Shanlin Liu [1, 2, 4, #] Chentao Yang[2, #], Chengran Zhou[2,5 #], Xin Zhou[1, 3, *]

1. Beijing Advanced Innovation Center for Food Nutrition and Human Health, China Agricultural University, Beijing 100193, People's Republic of China
2. BGI-Shenzhen, Shenzhen, 518083, China
3. College of Plant Protection, China Agricultural University, Beijing 100193, People's Republic of China
4. Centre for GeoGenetics, Natural History Museum of Denmark, University of Copenhagen, Copenhagen, Denmark
5. Key Laboratory of Bio-Resources and Eco-Environment, Ministry of Education, College of Life Sciences, Sichuan University, Chengdu 610000, China.

[#]Equal contribution
*Corresponding to: Xin Zhou

Abstract

Over the past decade, biodiversity scientists have dedicated tremendous efforts in constructing DNA reference barcodes for rapid species registration and identification. Although analytical cost for standard DNA barcoding has been significantly reduced since early 2,000, further dramatic reduction on barcoding costs is unlikely because the Sanger sequencing is approaching its limits in throughput and chemistry cost. Constraints in barcoding cost not only led to unbalanced barcoding efforts around the globe, but also refrained High-Throughput-Sequencing (HTS) based taxonomic identification from applying binomial species names, which provide crucial linkages to biological knowledge. We developed an Illumina-based pipeline, HIFI-Barcode, to produce full-length COI barcodes from pooled PCR amplicons generated by individual specimens. The new pipeline generated accurate barcode sequences that were comparable to Sanger standards, even for different haplotypes of the same species that were only a few nucleotides different from each other. Additionally, the new pipeline was much more sensitive in recovering amplicons at low quantity. The HIFI-Barcode pipeline successfully recovered barcodes from over 78% of the PCR reactions that didn’t show clear bands on the electrophoresis gel. Moreover, sequencing results based on the single molecular sequencing platform, Pacbio, confirmed the accuracy the HIFI-Barcode results. Altogether, the new pipeline can provide an improved solution to produce full-length reference barcodes at about 1/10 of the current cost, enabling construction of comprehensive barcode libraries for local fauna, leading to a feasible direction for DNA barcoding global biomes.

Background

Over the past decade, biodiversity research has seen paradigm shifts in methodology developments and applications [1], where standard DNA sequences, e.g., DNA barcodes, are adopted for fast and accurate taxonomic diagnoses, and High Throughput Sequencing (HTS) platforms are employed in analysis of complex biological samples, including bulk samples [2, 3], environmental DNA (eDNA, [4]), invertebrate-derived DNA (iDNA, [5, 6]) etc. DNA barcode reference libraries have been constructed globally via synergistic effort, resulting in well-curated, centralized barcode registration databases, e.g., the Barcode of Life Data systems [7], which has recently reached a milestone for 5-million barcodes, covering ca. 0.26 million species (accessed in July 2017). These DNA barcodes have been effectively facilitating species identification, phylogenetic reconstruction [8], and understanding of interspecific interactions and community structures [1].

Along with the rapid accumulation of global barcode references for various taxon groups, significant effort has been made in digitalizing biomes, e.g., sequencing all taxa of particular lineages found in entire range of national parks or islands [9]. Early efforts in barcoding biomes have employed standard Sanger sequencing-based approaches to characterizing focal fauna [10-12]. Alternatively, boosted by HTS technologies, DNA metabarcoding and mitochondrial metagenomics (mitochondrial genome skimming) have been applied in investigations of local biodiversity and in evaluation of biological managements [13-17]. These practices allow investigators to rapidly understand species richness or even approximation for species evenness and/or biomass for complex biological samples [4, 18]. A typical dilemma, however, is the lack of local barcode references, from which HTS biodiversity analysis could draw conclusions on species occurrences. This is primarily due to unbalanced barcoding efforts around the globe, where regions in desperate needs for biodiversity research are typically suffering from insufficient funding for taxonomy work, especially for DNA based studies. Consequently, HTS-

based taxonomic registrations are often constraint to applying Molecular Operational Units (MOTUs) instead of binomial species names, therefore unable to associate existing biological and ecological knowledge to the resultant diversity composition.

Admittedly, the analytical cost for standard DNA barcoding has been significantly reduced since early 2000, a result from the development of centralized and industrialized barcoding facilities and automated pipelines [1]. Currently, the average production cost for a reference barcode is ca. 10 USD, excluding that for sample collection and handling. Further dramatic reduction on barcoding costs is unlikely because Sanger sequencing technology is approaching its limits in throughput and associated chemistry cost. It is estimated that 100 million specimens would need to be sequenced to complete the global barcode registration [1], which translates into a roughly 1-billion-dollar budget for merely reference constructions. A similar challenge was seen in the sequencing of the first human genome, where an initial budget of over 3 billion USD was estimated based on the application of Sanger sequencing [19]. Thanks to the advent of HTS technologies over the past decade, the current cost for a human genome is within the range of just a few thousand USD, if not less.

An early study using HTS in generating barcodes from single specimens employed the Roche 454 platform [20], which was rapidly phased out due to limited throughput capacity (hence high chemistry cost). Illumina platforms (e.g., Hiseq and Miseq) have been primarily applied in recent practices [21]; but these are constraint by relatively short read lengths (100-300 bps). Even with the most recent Miseq model at 300bp paired-end (PE) sequencing, full-length barcodes (e.g., ~700 bps for COI including primers) are beyond the sequencing range. Therefore, existing pipelines are forced to produce a fragment of the standard barcodes (e.g., 313bp,[22]) or to apply 2 rounds of PCR amplifications, each targeting on a proportion of the full barcodes [21]. Obviously, full-length barcodes are desired for constructing barcode references and extra

amplification procedure should be avoided when possible for cost control and simplification of pipelines. In particular, efficient primers might be difficult to identify in the mid-COI barcode region across taxon groups. Alternatively, short HTS reads can be assembled into much longer scaffolds, which is a standard practice in *de novo* genome or transcriptome assembling. In fact, a specific assembly algorithm, SOAPBarcode, has been developed for recovering full-length barcodes from pooled arthropod samples [23].

Here, we introduce a more straightforward and cost-efficient HTS pipeline that generates full-length reference barcodes - HIFI-Barcode (Fig. 1). Briefly, individual genomic DNA was extracted separately and amplified on a 96-well plate using 96 sets of uniquely tagged primers. Amplicons were then pooled and sequenced on an Illumina Hiseq 4000 platform at 150 paired-end (PE). Mixed HTS reads were assembled using a customized bioinformatics pipeline to obtain barcode sequence for each individual. Compared to aforementioned studies [21, 22], our method can deliver standard full-length barcodes via a single PCR reaction and the sequencing is carried out on HiSeq platforms, the most cost-effective HTS platform currently available. Using Sanger barcodes as the gold standard, the new pipeline can generate accurate individual barcode sequences, even for haplotypes of the same species that are only a few nucleotides different from each other. Additionally, the new pipeline is much more sensitive in recovering amplicons at low quantity. Over 78% (25/32) of the "failed" PCR amplicons (those without clear bands on an electrophoresis gel) were successfully recovered at high-quality using the new pipeline. In addition, the single-molecule sequencing platform, Pacbio, has also been adopted in our study to evaluate the accuracy of the HIFI-Barcode method. Altogether, the new pipeline can provide an alternative solution to produce full-length reference barcodes at about 1/10 of the current cost, enabling larger-scale biodiversity barcoding initiatives, especially for areas where DNA references are scarce.

Material and methods

1. DNA preparation

Insect specimens were collected in Laohegou Natural Reserve, Sichuan Province, China. Genomic DNA was extracted in an independent study using the Glass Fiber Plate method following manufacturer's protocol [24]. Two 96-well plates were prepared for the current work: one plate containing 96 high-quality lepidopteran DNA (showing clear band of standard DNA barcode amplicon on an electrophoresis gel) is used to evaluate the accuracy of our HTS method using Sanger barcodes as the gold standard; a second plate containing 95 randomly selected DNA (mostly dipterans) regardless of quality and PCR yields plus a blank control is prepared to examine the success rate of our HTS method compared with the classic Sanger approach.

2. DNA amplification and sequencing

Ninety-six pairs of different tags were added to both ends of a common COI barcode primer set (LCO1490 and HCO2198, Supplemental Table S1) [25], with each tag containing 5 bps allowing for ≥ 3 bp differences from each other. Each PCR reaction contained 1 μl of DNA template, 16.2 μl of molecular biology grade water, 3 μl of 10X reaction buffer (Mg2+ plus), 2.5 μl of dNTPs mix (10 mM), 1 μl of forward and reverse primers (10 mM), and 0.3 μl of TaKaRa Ex Taq polymerase (5 U/μl). The amplification program included a touchdown thermocycling profile of 94°C for 1 min, 5 cycles of 94°C for 30 sec, 45°C for 40 sec, and an extension at 72°C for 1 min, followed by 35 cycles of 94°C for 30 sec, 51°C for 40 sec, and 72°C for 1 min, with a final extension at 72°C for 10 min, and finally holding at 12°C. All amplicons were visualized on a 1.2% 96 Agarose E-gel (Biowest Agarose). All PCR products from each plate were pooled using 1 μl per sample resulting in two 96 μl mixtures, which were sent to BGI and sequenced using a Hiseq 4000. PCR amplicons were fragmented to construct library of an insert-size of 250 bp and sequenced with a strategy of 150 PE. A second set of PCR mixture of the 2nd plate (576μl, 6 μl per sample) was sequenced using PacBio RS II at NextOmics.

3. HIFI-Barcode assembly

Data filtering: Reads of bad-quality were removed from raw data: 1) reads with adapter contamination (≥15 bps alignment length and ≤3 mismatches); 2) reads with >10 Ns; 3) reads with >50 bps of low quality (Phred quality score = 2, ASCII 35 "B", Illumina 1.8+ Phred+33).

Read assignment: Firstly, reads containing 5' and 3' ends of each individual were identified based on their unique 5-mer tags and corresponding primer sequences using in house Perl scripts. Then, for each individual, identical reads were clustered to obtain unique 5' and 3' sequences. Each individual may contain multiple unique terminal sequences at varied abundances due to haplotype heterogeneity (mitochondrial heteroplasmy) or artefacts (PCR or sequencing errors). Next, the most abundant unique sequence was chosen for the following overlapping and assembly procedures. In addition, if the next most abundant unique sequence had an abundance ≥ 1/10 of that of the most abundant unique sequence at <98% similarity, it was also retained to confirm identities, e.g., parasites, *Wolbachia*, gut contents that were co-amplified in PCR. After that, corresponding pairs of the afore-chosen reads were identified according to their titles and then paired-end reads were overlapped using COAP [26] with an identity cutoff of 95%. Overlapped reads could vary in sequence length due to insert-size fluctuation during ultrasonic shearing. Thus, consensus 5' and 3' sequences of each individual were achieved using in house Perl scripts where ends with read coverage < 5 were trimmed off (Fig. 2).

Gap filling: Algorism adopted from SOAPBarcode [23] (Supplemental Fig. S1) was applied to fill the gaps between 5' and 3' terminal scaffolds of each individual to complete the full-length barcodes. Briefly, for each individual, the 5' end was defined as the start point, and the 3' end as the end point. Then, the kmer set from de brujin graph was walked step by step from the start point to

the end point to find potential assembly paths. Several strategies were applied to ensure correct paths: 1) kmers of abundance < 10% of the average kmer abundance before path bifurcation were removed; 2) if there was more than one out degree remaining after step one, common reads were counted between different out degrees and the kmer located before the last bifurcation, and the out degrees of common reads < 10% of the average abundance were removed; 3) paths expanding beyond the pre-set length (standard COI barcode length plus primers) without an end point were removed.

5. Data filtering and read assignment for Pacbio

Pacbio SmrtAnalysis pipeline (https://github.com/PacificBiosciences) was adopted to extract 28,770 circular consensus sequencing (CCSs) from 1.1G raw data. Then, CCSs of ≥ 15 passes were chosen for next steps: 1) 22,075 CCSs were demultiplexed by their corresponding indices using an in-house Perl script, allowing a maximum of 1bp deletion at the 5' end of forward index or the 3' end of reverse index. 2) for each sample, sequences with a length range out of 658 ± 6bp were removed and the remaining unique sequences were sorted by pass numbers and identical sequences were clustered together; 3) unique sequence of the most abundant cluster was retained as the correct barcode sequence for each sample.

6. Comparisons between HTS, Sanger barcodes and Pacbio clusters

Barcode sequences obtained by Sanger, HIFI-Barcode method and Pacbio were subject to phylogenetic tree constructions using MEGA7 (Neighbor-joining and 1,000 bootstrap) and iTOL [28]. BWA [29] was applied to align raw reads to assembled HTS barcodes to examine discrepancies between HTS and Sanger sequences.

Results

A total of 4,824,443 and 4,439,345 PE reads for the 1st and 2nd plate were

obtained after data filtering, respectively, using Hiseq 4000.

For the 1st plate, a total of 1,910,616 (39.60%) reads were assigned to their corresponding samples as either 5' or 3' end, and 1,898,372 (39.34%) as reads belonging to intermediate regions, while 1,015,455 (21.05%) reads were identified as primer dimers or short PCR chimeras. The abundance of end reads for each sample varies significantly, ranging from 2,444 to 64,705. After clustering at 100%-similarity for the 5' and 3' end reads, most samples (61 out of 96) obtained single unique reads after read assignment. The 2nd plate possessed similar read distribution and details of both plates were summarized in Table 1.

One cell of Pacbio data containing 28,770 circular consensus sequencing (CCS) from 1,201,158 raw reads were generated for the 2nd plate. CCS reads had an average pass number of 26.5 and were assigned to 82 samples after demultiplexing. (Table 1). Note that a single Pacbio sequencing read can reach as long as 40 kb. Therefore, short CCS read of high quality can be sequenced dozens of times, which in turn effectively corrects sequence errors associated with the platform [30].

Accuracy and Efficiency:

Sanger barcodes were obtained from all 96 lepidopteran samples of the 1st plate (Fig. 3A), including 91 haplotypes and 85 OTUs using a similarity threshold of ≥98%. The HIFI-Barcode assemblies were successful for all 96 samples and showed high accuracy compared to Sanger sequences. Even identical or highly similar barcodes from individuals of the same species were correctly assembled, e.g., A2 versus F7, B1 versus E1, and C7 versus G4 (Fig. 3B and 3C). A total of 43 ambiguous sites (out of 63,168 bps) found in Sanger barcodes were identified to a specific nucleotide in HIFI barcodes (e.g., Fig. 3D, Fig. 4B). Only 9 HIFI barcodes showed a single nucleotide difference from the corresponding Sanger sequences, which could reflect ambiguous base-calling in Sanger sequencing or genuine heteroplasmy in the examined individual. At

least two of the discrepancies were proven to be heterozygotes via mapping raw reads against discrepant sites (Fig. 4A).

In the 2nd plate, samples were randomly selected regardless of their DNA quality and PCR success rates. Sixty-three PCR reactions showed clear bands on the electrophoresis gel (Supplemental Table S2), of which 62 resulted in Sanger barcodes. The HIFI-Barcode pipeline successfully produced full-length HTS sequences for all 62 corresponding Sanger barcodes at high accuracy (56 at 100% match, 5 with 1 mismatch, and 1 with 3 mismatches, Supplemental Fig. S2). In addition, HIFI barcodes were successfully generated from 25 out of the 32 PCR amplicons that had no clear bands (Supplemental Table S2), increasing the overall success rate from 66.32% to 92.63%, for the Sanger and HIFI-Barcode methods, respectively (Fig. 5). To further evaluate the accuracy of the newly developed HIFI-Barcode pipeline especially for those where PCR reactions failed, we also sequenced pooled PCR amplicons using Pacbio. The CCSs used in our study had pass numbers > 15, which meant the same molecule was sequenced repeatedly for more than 15 times. Thus the consensus nucleotides for each sequence were corrected from sequencing errors associated with the platform (ca. 10% on average). The overall success rates for Pacbio was 86.32%. Of the 25 HIFI barcodes where Sanger failed, 18 Pacbio barcodes were obtained. Among these, 10 were identical to the corresponding HIFI barcodes; 3 had 1 or 2 sites matched with one of the two heterozygous alleles from HIFI barcodes; and 5 showed errors in amino acid translation (e.g., stop codon) possibly due to sequencing errors in Pacbio (Supplemental Table S3 and Supplemental file S1).

Non-target sequences detected by HIFI-Barcode

During the HIFI-Barcode assembly procedure, terminus sequences with ≥1/10 abundance of that of the most abundant scaffolds at <98% similarity were retained for assembly and identity check. This analysis allowed detection of 18 non-target sequences co-amplified from the 2 plates (Supplemental Table S4),

in addition to COI barcodes. Cross examinations against both NCBI and barcode sequences from the focal plates suggested origins including *Wolbachia* (2), fungus (1), cross-contamination from adjacent wells (7), as well as potential PCR errors and pseudo-genes (8). The presence of non-target PCR products from the 2nd plate were further confirmed by Pacbio sequencing at >99% identity, therefore ruling out the likelihood of assembly errors in the HIFI-Barcode pipeline. These low-quantity sequences are likely common in regular PCR-based pipelines and detectable by HTS-based approaches. But they can be easily filtered out from genuine COI barcodes following the pipeline described in this study.

Discussion

It is widely acknowledged that we have been undergoing unprecedented global biodiversity loss [31]. DNA-based approaches, e.g., DNA barcoding, DNA metabarcoding, mitochondrial metagenomics (mitochondrial genome skimming), have demonstrated efficacy in accelerating biodiversity inventories of large geographical ranges. These standardized and largely automated procedures will provide pivotal information to understand how biodiversity loss is characterized and how to desist from it. New methodologies enable rapid collection of biodiversity and ecology data at large scale over space and time, which in-turn benefits policy-makers at varied management levels and research groups [32].

Interpreting molecular results using existing knowledge on biology, ecology and evolution would require a linkage between DNA references and Linnaeus names, which is one of the fundamental roles of DNA barcoding initiatives. The construction of comprehensive barcode references is still, to a large extent, expensive and sometimes prohibitive. This is particularly true for studies targeting on a wide range of taxa from a large area of natural habitat. Although the most represented DNA barcode database (BOLD) now hosts barcodes for 0.26 million species, accounting for ca. 1/4 of described species, chances of

encountering a novel barcode are still very high, especially for many biodiversity hotspots. Even if an ecological study focuses on just a small proportion of the focal diversity, it is not uncommon that hundreds to thousands of species would need to be barcoded to draw meaningful conclusions. In addition, multiple individuals of the same species (ideally from distinct populations) would need to be sequenced to reflect intraspecific genetic diversities. There is no consensus on the ideal number of conspecific individuals to be sequenced, but in practice an average of 10 is often followed, while some study recommends 20 [33], if not a lot more. Therefore, roughly tens of thousands of individuals, requiring hundreds of thousands of USD, are expected to suffice a regular ecology study, just for the molecular analysis (for a recent example, please see [12]). While the HTS-based approaches have shown promising power in analyzing complex sample mixtures at much reduced unit cost [2-4, 14], one would still need to establish DNA barcode references to be able to go beyond OTU-based interpretation.

The HIFI-Barcode method, as the results showed, offers a novel route to produce mass volumes of reliable barcode sequences at significantly reduced cost. The main costs of the HIFI-Barcode pipeline include consumable chemistries, library construction, high-throughput sequencing, and informatics. Despite the increased one-time cost in ordering multiple unique sets of primers, the cost on primers per unit reaction is negligible. Following our protocols, the average cost for a HIFI barcode is around 1 USD, as opposed to 10-20 USD in the standard Sanger approach. Further saving on the production cost is achieved by increased success rates, especially for amplicons with low quantity. In our test, ca. 1/3 of the 2nd plate would have been re-amplified in standard barcoding protocols, using a different set of primers, followed by gel examination, positive picking, PCR purification, and Sanger-sequencing.

By complementing the barcode reference library at <1/10 of the current cost, the new approach also reinforces rapid constructions of organelle genomes, e.g., mitochondria and chloroplasts. A number of pilot studies have

demonstrated that full mitochondrial genomes can provide elevated power in bulk sample analysis [18, 34]. New approaches to assembling full mito-genomes or the majority of the coding genes have been developed for shotgun sequencing of individual specimens [35], pooled taxa [18, 34], and transcriptomes [35]. In particular, mito-genome assembly through direct shotgun sequencing of mixed taxa can significantly reduce the library construction cost for HTS. Bait sequences, which regularly includes standard COI barcodes, are important for assigning mixed mitochondrial scaffolds to a specific taxon. This is critical especially if the phylogenetic signal of the scaffolds alone is not sufficient to attribute assemblies to species, e.g., when multiple closely related species are pooled. In fact, having multiple bait sequences per species will significantly remove bioinformatics challenge during the assembly procedure [36], which now becomes financially feasible with the help of the HIFI-Barcode pipeline.

Several aspects can be further improved for our method: 1) Multiple barcode markers (e.g., COI, CYTB, 12S, etc.) can be pooled in a single shotgun sequencing effort without increasing tag complexity, which will again alleviate analytical cost. 2) The pooled PCR amplicons were subject to library construction directly in the present study. The proportion of primer dimers and short PCR chimera reached as high as ca. 21% in our raw reads, which can be easily removed using size-preference magnetic beads. 3) Addition of inosine to the 3' terminus of the primer may increase its universality and will further elevate the successful rate and efficiency. 4) Longer tags allowing for pooling more individuals (e. g. 384-well plate) can further increase the throughput capacity.

In summary, the HIFI-Barcode method provides a HTS-based approach with improved economic efficiency, which allows investigators to produce standard full-length barcodes at ca. 1/10 of the current cost. The new protocol not only generates barcode sequences of high quality that are comparable to Sanger-barcodes, but also increases overall sequencing success rates by detecting

PCR amplicons in minute quantities. This new method enables construction of comprehensive barcode libraries for local fauna, leading to a feasible direction for DNA barcoding global biomes.

Availability of source code and requirements

- Project name: HIFI – Barcode project
- Operating system(s): Unix, Linux
- Programming language: PERL
- Other requirements: GCC version ≥ 4.4.5
- License: GNU General Public License version 3.0 (GPLv3)
- Any restrictions to use by non-academics: none

Availability of Supporting Data

Availability of supporting data and materials code and data are available in the GigaScience GigaDB repository (XXX). Source code also can be found in https://github.com/comery/HIFI-barcode-hiseq and https://github.com/comery/HIFI-barcode-pacbio.

Abbreviations

BOLD - the Barcode of Life Data systems; CCSs – circular consensus sequencing; eDNA – environmental DNA; HTS – High Throughput Sequencing; GB – Gigabase; iDNA – invertebrate-derived DNA; MOTUs – Molecular Operational Units; PCR - Polymerase Chain Reaction; PE – Paired End.

Acknowledgements

We thank Dr. Ding Yang from China Agricultural University for contributing samples and Yongfei Du from BGI-Shenzhen for his help on coordinating the HiSeq sequencing of our 2nd plate. We would like to thank Min Tang, Guanliang Meng for assistant in figure preparation. This work is supported by the China National GeneBank, BGI and by China Agricultural University through the

Chinese Universities Scientific Fund, 2017QC114 to XZ.

Author contributions:

X.Z. and S.L. designed the study; S.L. coordinated the project and led the analyses; C.Z. and C.Y. led the bench work and contributed to the analyses; S.L., C.Z., and C.Y. formulated the early drafts, and X.Z. revised the manuscript.

Competing interests

The authors declare that they have no competing interests.

1. Hebert PD, Hollingsworth PM, Hajibabaei M: **From writing to reading the encyclopedia of life**. *Phil Trans R Soc B* 2016, **371**(1702):20150321.
2. Yu DW, Ji YQ, Emerson BC, Wang XY, Ye CX, Yang CY, Ding ZL: **Biodiversity Soup: Metabarcoding of arthropods for rapid biodiversity assessment and biomonitoring**. *Methods in Ecology and Evolution* 2012, **3**(4):613-623.
3. Zhou X, Li Y, Liu S, Yang Q, Su X, Zhou L, Tang M, Fu R, Li J, Huang Q: **Ultra-deep sequencing enables high-fidelity recovery of biodiversity for bulk arthropod samples without PCR amplification**. *GigaScience* 2013, **2**(1):4.
4. Taberlet P, Coissac E, Hajibabaei M, Rieseberg LH: **Environmental DNA**. *Molecular Ecology* 2012, **21**:1789–1793.
5. Calvignac-Spencer S, Merkel K, Kutzner N, Kühl H, Boesch C, Kappeler PM, Metzger S, Schubert G, Leendertz FH: **Carrion fly-derived DNA as a tool for comprehensive and cost-effective assessment of mammalian biodiversity**. *Molecular Ecology* 2013, **22**(4):915-924.
6. Schnell IB, Thomsen PF, Wilkinson N, Rasmussen M, Jensen LR, Willerslev E, Bertelsen MF, Gilbert MTP: **Screening mammal biodiversity using DNA from leeches**. *Current biology* 2012, **22**(8):R262-R263.
7. Ratnasingham S, Hebert PDN: **BOLD: The Barcode of Life Data System (http://www. barcodinglife. org)**. *Molecular ecology notes* 2007, **7**(3):355-364.
8. Zhou X, Frandsen PB, Holzenthal RW, Beet CR, Bennett KR, Blahnik RJ, Bonada N, Cartwright D, Chuluunbat S, Cocks GV: **The Trichoptera barcode initiative: a strategy for generating a species-level Tree of Life**. *Phil Trans R Soc B* 2016, **371**(1702):20160025.
9. Miller SE, Hausmann A, Hallwachs W, Janzen DH: **Advancing taxonomy and bioinventories with DNA barcodes**. *Phil Trans R Soc B* 2016, **371**(1702):20150339.
10. Zhou X, Robinson JL, Geraci CJ, Parker CR, Flint Jr OS, Etnier DA, Ruiter D, DeWalt

RE, Jacobus LM, Hebert PD: **Accelerated construction of a regional DNA-barcode reference library: caddisflies (Trichoptera) in the Great Smoky Mountains National Park**. *Journal of the North American Benthological Society* 2011, **30**(1):131-162.

11. Zhou X, Adamowicz SJ, Jacobus LM, DeWalt RE, Hebert PD: **Towards a comprehensive barcode library for arctic life-Ephemeroptera, Plecoptera, and Trichoptera of Churchill, Manitoba, Canada**. *Frontiers in zoology* 2009, **6**(1):1.
12. Telfer AC, Young MR, Quinn J, Perez K, Sobel CN, Sones JE, Levesque-Beaudin V, Derbyshire R, Fernandez-Triana J, Rougerie R: **Biodiversity inventories in high gear: DNA barcoding facilitates a rapid biotic survey of a temperate nature reserve**. *Biodiversity data journal* 2015(3).
13. Valentini A, Taberlet P, Miaud C, Civade R, Herder J, Thomsen PF, Bellemain E, Besnard A, Coissac E, Boyer F: **Next-generation monitoring of aquatic biodiversity using environmental DNA metabarcoding**. *Molecular Ecology* 2016.
14. Hajibabaei M, Shokralla S, Zhou X, Singer GAC, Baird DJ: **Environmental barcoding: a next-generation sequencing approach for biomonitoring applications using river benthos**. *PloS one* 2011, **6**(4):e17497.
15. Coissac E, Riaz T, Puillandre N: **Bioinformatic challenges for DNA metabarcoding of plants and animals**. *Molecular Ecology* 2012, **21**:1834–1847.
16. Bienert F, Dedanieli S, Miquel C, Coissac E, Poillot C, Brun JJ, Taberlet P: **Tracking earthworm communities from soil DNA**. *Molecular Ecology* 2012, **21**(8):2017-2030.
17. Baird DJ, Hajibabaei M: **Biomonitoring 2.0: a new paradigm in ecosystem assessment made possible by next-generation DNA sequencing.**. *Molecular Ecology* 2012, **21**(8):2039-2044.
18. Gillett CP, Crampton-Platt A, Timmermans MJ, Jordal B, Emerson BC, Vogler AP: **Bulk de novo mitogenome assembly from pooled total DNA elucidates the phylogeny of weevils (Coleoptera: Curculionoidea)**. *Molecular biology and evolution* 2014:msu154.
19. Krulwich R, LANDER E: **Cracking the Code of Life**. In.: Public Broadcasting Service; 2001.
20. Shokralla S, Gibson JF, Nikbakht H, Janzen DH, Hallwachs W, Hajibabaei M: **Next-generation DNA barcoding: using next-generation sequencing to enhance and accelerate DNA barcode capture from single specimens**. *Molecular Ecology Resources* 2014, **14**(5):892-901.
21. Shokralla S, Porter TM, Gibson JF, Dobosz R, Janzen DH, Hallwachs W, Golding GB, Hajibabaei M: **Massively parallel multiplex DNA sequencing for specimen identification using an Illumina MiSeq platform**. *Scientific reports* 2015, **5**.
22. Meier R, Wong W, Srivathsan A, Foo M: **$1 DNA barcodes for reconstructing complex phenomes and finding rare species in specimen‐rich samples**. *Cladistics* 2016, **32**(1):100-110.

23. Liu S, Li Y, Lu J, Su X, Tang M, Zhang R, Zhou L, Zhou C, Yang Q, Ji Y: **SOAPBarcode: revealing arthropod biodiversity through assembly of Illumina shotgun sequences of PCR amplicons**. *Methods in Ecology and Evolution* 2013, **4**(12):1142-1150.

24. Ivanova NV, Dewaard JR, Hebert PD: **An inexpensive, automation-friendly protocol for recovering high-quality DNA**. *Molecular Ecology Resources* 2006, **6**(4):998-1002.

25. Vrijenhoek R: **DNA primers for amplification of mitochondrial cytochrome c oxidase subunit I from diverse metazoan invertebrates**. *Molecular marine biology and biotechnology* 1994, **3**(5):294-299.

26. Liu B, Yuan J, Yiu SM, Li Z, Xie Y, Chen Y, Shi Y, Zhang H, Li Y, Lam TW: **COPE: an accurate k-mer-based pair-end reads connection tool to facilitate genome assembly**. *Bioinformatics* 2012, **28**(22):2870-2874.

27. Kumar S, Stecher G, Tamura K: **MEGA7: Molecular Evolutionary Genetics Analysis version 7.0 for bigger datasets**. *Molecular biology and evolution* 2016, **33**(7):1870-1874.

28. Letunic I, Bork P: **Interactive tree of life (iTOL) v3: an online tool for the display and annotation of phylogenetic and other trees**. *Nucleic Acids Research* 2016:gkw290.

29. Li H, Durbin R: **Fast and accurate short read alignment with Burrows–Wheeler transform**. *Bioinformatics* 2009, **25**(14):1754-1760.

30. Eid J, Fehr A, Gray J, Luong K, Lyle J, Otto G, Peluso P, Rank D, Baybayan P, Bettman B: **Real-time DNA sequencing from single polymerase molecules**. *Science* 2009, **323**(5910):133-138.

31. Pimm SL, Jenkins CN, Abell R, Brooks TM, Gittleman JL, Joppa LN, Raven PH, Roberts CM, Sexton JO: **The biodiversity of species and their rates of extinction, distribution, and protection**. *Science* 2014, **344**(6187):1246752.

32. Díaz S, Demissew S, Carabias J, Joly C, Lonsdale M, Ash N, Larigauderie A, Adhikari JR, Arico S, Báldi A: **The IPBES Conceptual Framework—connecting nature and people**. *Current Opinion in Environmental Sustainability* 2015, **14**:1-16.

33. Luo A, Lan H, Ling C, Zhang A, Shi L, Ho SY, Zhu C: **A simulation study of sample size for DNA barcoding**. *Ecology and Evolution* 2015, **5**(24):5869-5879.

34. Tang M, Tan M, Meng G, Yang S, Su X, Liu S, Song W, Li Y, Wu Q, Zhang A: **Multiplex sequencing of pooled mitochondrial genomes—a crucial step toward biodiversity analysis using mito-metagenomics**. *Nucleic Acids Research* 2014, **42**(22):e166-e166.

35. Tan M, Zhang R, Xiang C, Zhou X: **The complete mitochondrial genome of the pumpkin fruit fly, Bactrocera tau (Diptera: Tephritidae)**. *Mitochondrial DNA Part A* 2016, **27**(4):2502-2503.

36. Timmermans MJ, Dodsworth S, Culverwell C, Bocak L, Ahrens D, Littlewood DT, Pons J, Vogler AP: **Why barcode? High-throughput multiplex sequencing of mitochondrial genomes for molecular systematics**. *Nucleic acids research* 2010, **38**(21):e197-e197.

Table 1. Read distribution of both Illumina and Pacbio platforms

	Raw read	Clean read	5' and 3' read	Read in-between	Recovered Indices	Sample size [1]	Single unique [2]	Full-length barcodes
Hiseq 1	8,567,336	4,824,443	1,910,616	1,898,372	96	39,805 (64,705; 2,444)	61	96
Hiseq 2	11,531,498	4,439,345	1,306,054	2,676,915	96	27,210 (101,512; 279)	45	88
Pacbio 2[*]	1,201,158	28,770	26.4	17,102	82	208 (1,696; 1)	NA	82
		**Total number [3]**	**Average pass [3]**	**Assigned [3]**				

Note: * number 1 and 2 in this column represent plate ID; 1. Read number possessed by samples in format as: average (max; min); 2. Number of clusters that left only 1 single representative candidate after read assignment filtering; 3. Statistics of Circular Consensus Sequence (CCS)

Figure legends

Figure 1. Schematic of HIFI-Barcode pipeline.

Figure 2. HIFI-Barcode assembly pipeline.

Figure 3. Comparison between HIFI-Barcode and Sanger reference. 3A, success rates of the 1st plate. For all 96 samples, both Sanger (left semicircle) and HIFI-Barcode (right semicircle) are successful in producing a full-length COI barcode. Samples with red out lining are marked on the phylograms; 3B, phylogenetic tree of all HIFI barcodes and Sanger references; 3C, close-up view of representative individuals; 3D, degenerate sites of Sanger references were recuperated by HIFI barcodes.

Figure 4. Discrepancies between Sanger and HIFI barcodes in the 1st plate, showing potential heterozygotes (4A) and differences between ambiguous Sanger base-calling and specific nucleotide identify in HIFI barcodes (4B).

Figure 5. Success rates of the 2nd plate. For each sample, the upper, left and right pies represent PCR, HIFI-Barcode and Pacbio, respectively. Gray represents failure and the others represent success.

Additional Files

Supplemental Figure S1. Algorism described in SOAPBarcode pipeline.

Supplemental Figure S2. Phylogenetic tree of samples sharing Sanger references, HIFI barcodes and Pacbio barcodes.

Supplemental Table S1. Indexed Primer sequences.

Supplemental Table S2. PCR electrophoresis results.

Supplemental Table S3. Comparison of 18 Pacbio barcodes and HIFI-barcodes

Supplemental Table S4. Non-target sequences detected by HIFI-Barcode

Supplemental File S1. Results of HIFI-barcode

Figure 1

Click here to download Figure fig1.pdf

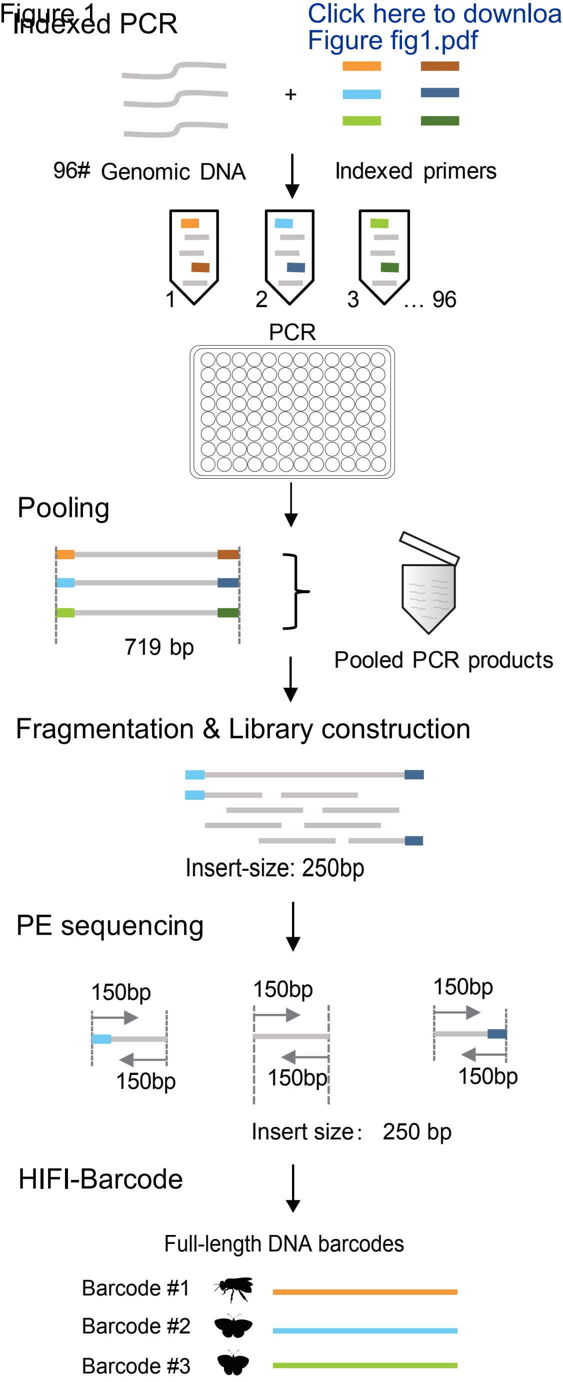

Figure 2

Click here to download Figure fig2.pdf

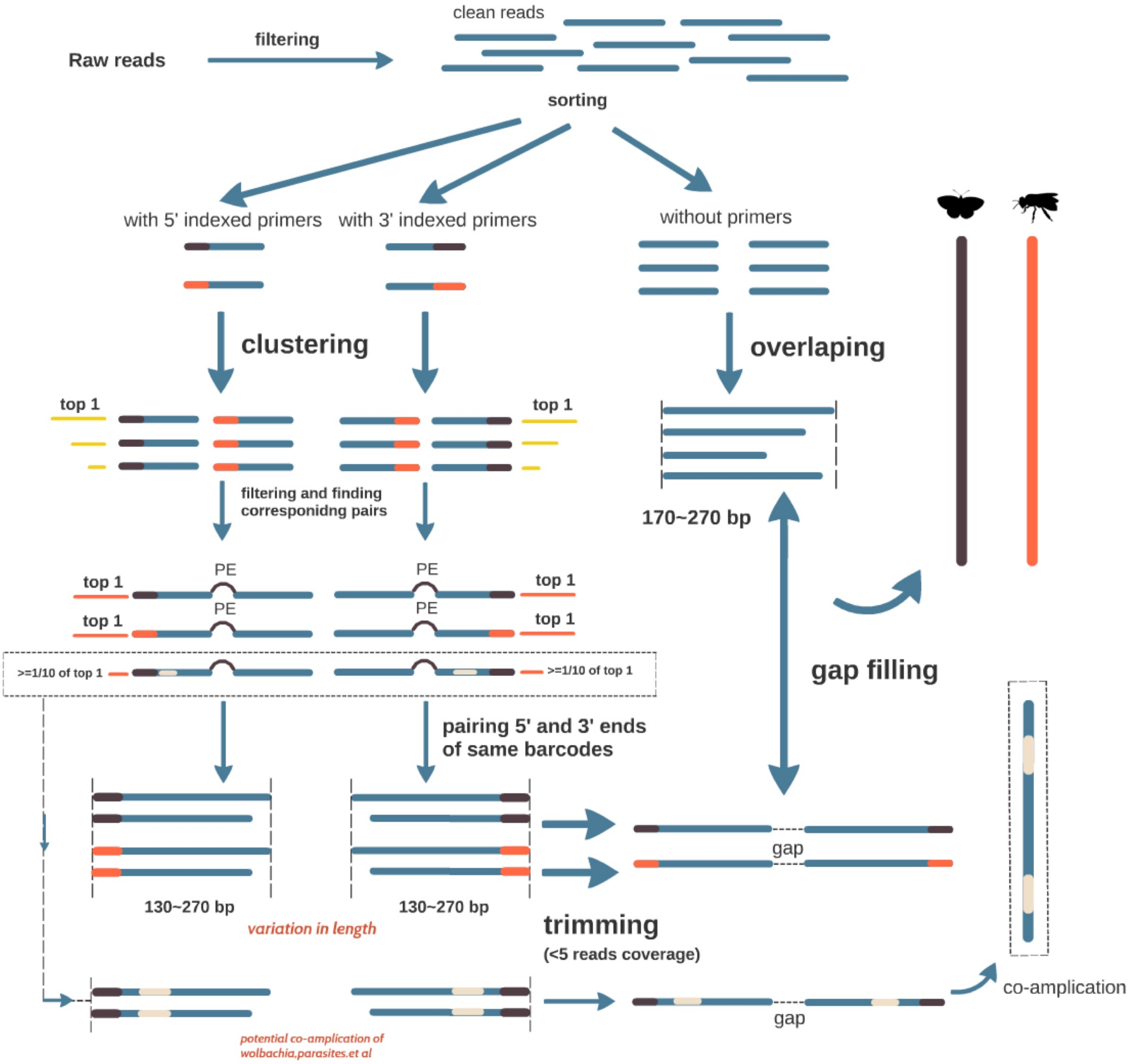

Figure 3

Click here to download Figure fig3.pdf

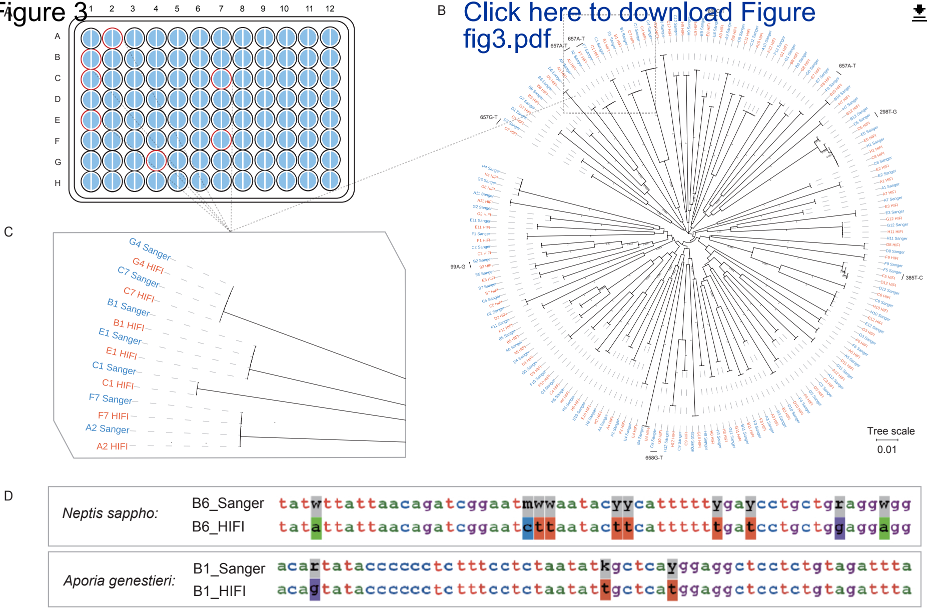

Figure 4

Click here to download Figure fig4.pdf

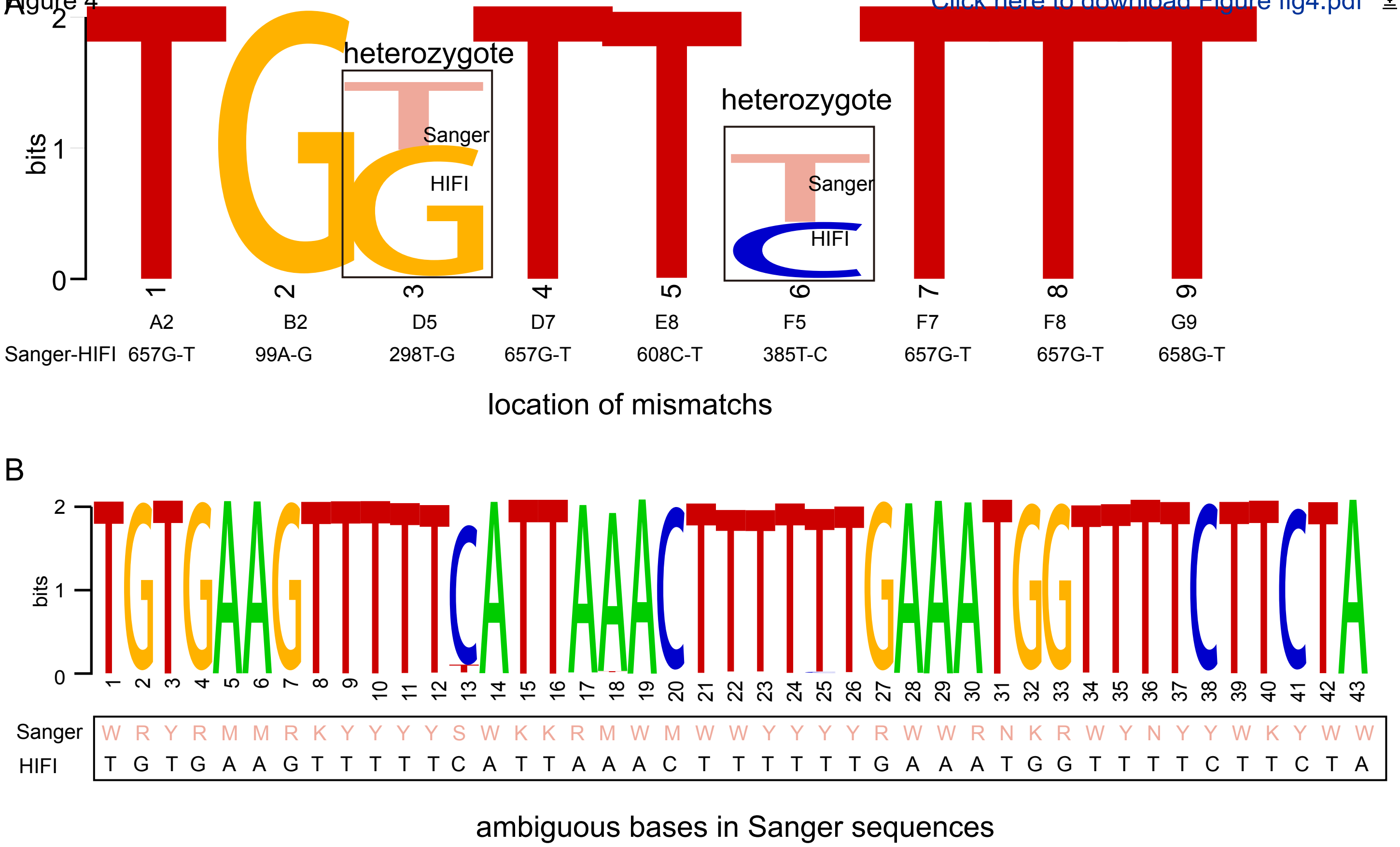

Figure 5

Click here to download Figure fig5.pdf

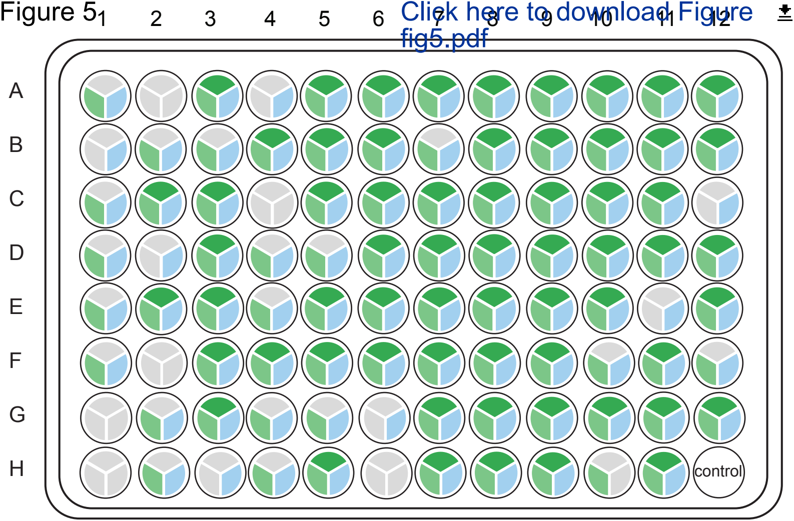

Supplementary table s1

Click here to access/download
Supplementary Material
supplementary table S1.xlsx

Supplementary table s2

Click here to access/download
Supplementary Material
supplementary table S2.xlsx

Supplementary table s3

Click here to access/download
Supplementary Material
supplemental table S3.xlsx

Supplementary table s4

Click here to access/download
Supplementary Material
supplemental table S4.xlsx

Supplementary figure s1

Click here to access/download
Supplementary Material
supplementary figure S1.png

Supplementary figure s2

Click here to access/download
Supplementary Material
supplemental figure S2.pdf

Click here to access/download
Supplementary Material
Supplemental file S1. Results of HIFI-barcode.rar
