# Supplementary figures and images for: Filling reference gaps via assembling DNA barcodes using high-throughput sequencing—moving toward barcoding the world
Source: Gigascience. 2017 Oct 25;6(12):1–8. doi: 10.1093/gigascience/gix104 (PMC5726475; doi:10.1093/gigascience/gix104)

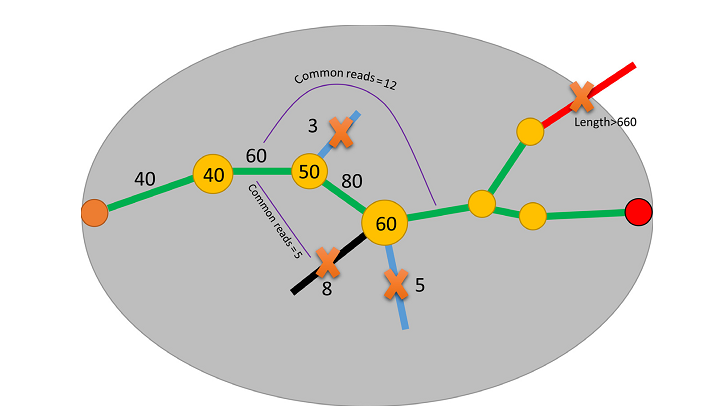

Supplement: Supplementary Figure S1. Algorithm described in the SOAPBarcode pipeline [file gix104_supplementary_figure_s1.png]

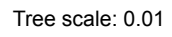

Supplement: Supplementary Figure S2. Phylogenetic tree of samples sharing Sanger references, HIFI-Barcodes, and Pacbio barcodes [file gix104_supplemental_figure_s2.pdf]

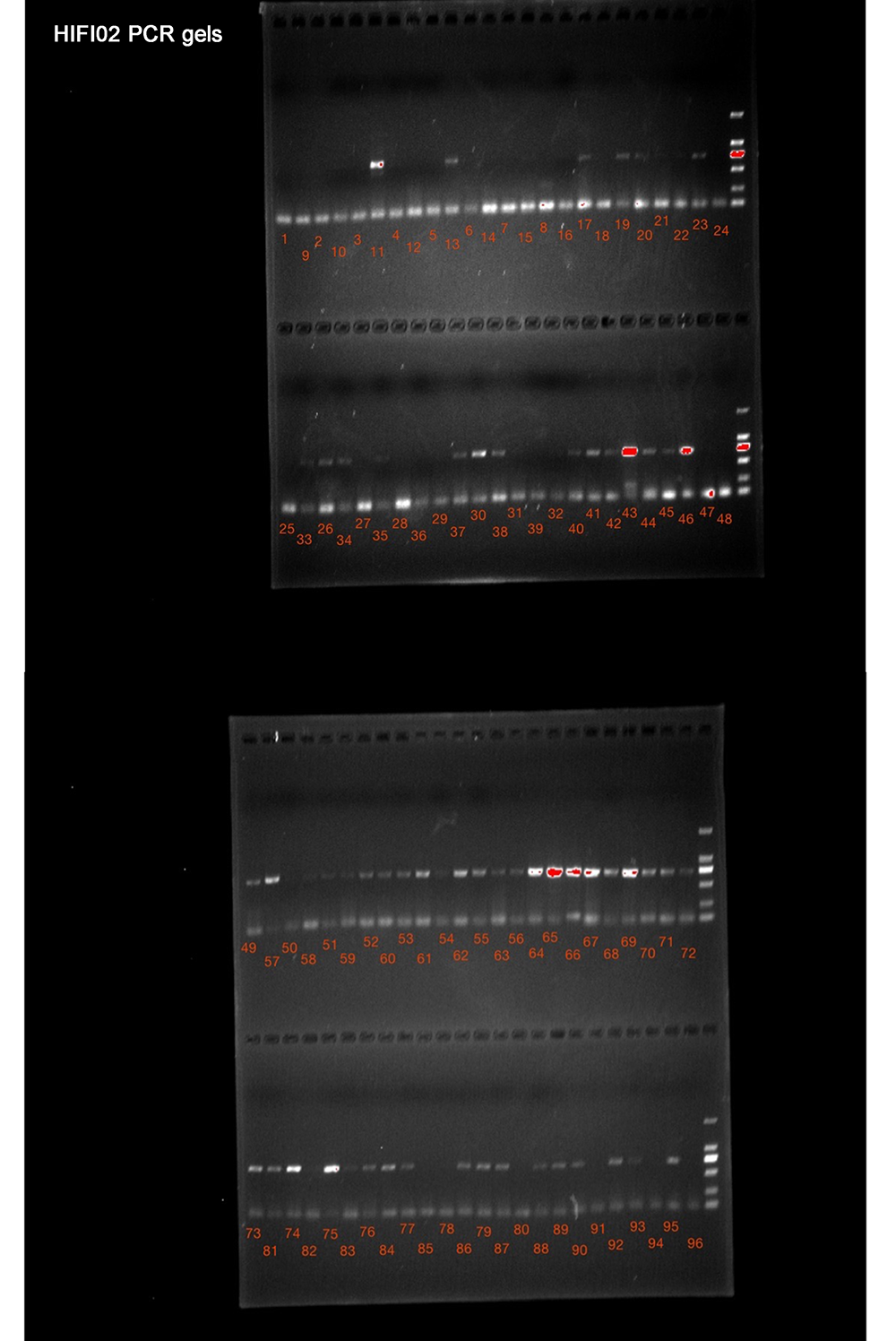

Supplement: Supplementary Figure S3. PCR electrophoresis results of the second plate [file gix104_supplemental_figure_s3.png]
