# Supplementary material for: Filling reference gaps via assembling DNA barcodes using high-throughput sequencing—moving toward barcoding the world
Source: Gigascience. 2017 Oct 25;6(12):1–8. doi: 10.1093/gigascience/gix104 (PMC5726475; doi:10.1093/gigascience/gix104)
Supplement: gix104_Response-to-Reviewer-Comments_Original-Submission.pdf [file gix104_response-to-reviewer-comments_original-submission.pdf]

Author's Response To Reviewer Comments

Dear Scott,

We have carefully revised the manuscript in accordance with reviewers' comments. Enclosed please find our corresponding responses in detail. We have addressed all issues raised by the reviewers. Thanks to the constructive suggestions from both reviewers, we think our manuscript has been significantly improved. We sincerely hope this manuscript is now suitable for publication with GigaScience. Thank you for all your help and looking forward to hearing from you soon.

Sincerely yours,
Xin Zhou

Please find the following responses to editorial comments:

Reviewer comments:

Reviewer #1: The manuscript describes a novel method to recover full COI barcodes for individual specimens using high throughput sequencing technology for construction of reference databases linking species names and the barcode sequences, which are crucial for assessments of biodiversity based on molecular methods like metabarcoding.
The method overcomes a problem of Illumina platform (i.e. length of reads [typically150+150 bp] is short for standard COI barcode [>600 bp]). Compared with previously published method for the same purpose (ref.21 Shokralla et al. 2015), the method was improved in cost effectiveness, thanks to the simplified laboratory protocol and superb bioinformatics procedures, although one of the basic ideas (gap-filling) were already published (ref. 23). Further, the authors validated their method by using Pacbio, another (expensive) sequencing platform.
The paper will contribute to studies on biodiversity and meets the scope of the journal. I recommend it be accepted for publication after minor revision.

- Please clarify in the figure legend that sequence logos shown in Fig 4 were based on alignments of Illumina raw reads onto assembled HIFI-barcodes.
Response: Thanks for your comments. We have modified the legend of Fig. 4 to supplement the alignment info – "Entropy weight was calculated based on the strength of read depth by aligning Illumina raw reads onto assembled HIFI-barcodes".

- What does "heterozygote" mean for mitochondrial genes? Do you mean heteroplasmy?
Response: Thank you for your kindly remind. We have changed it to heteroplasmy.

- I would suggest the authors talk potential co-amplification of nuclear mitochondrial pseudogenes (so-called numts) by PCR.
Response: We appreciate your suggestion for the pseudogenes which has been pointed out in line 5, page 11 – "as well as potential PCR errors and pseudo-genes (numts)". We have also added another sentence in line 8, page 11, - "therefore ruling out the likelihood of assembly errors in the HIFI-Barcode pipeline, suggesting they are co-amplified numts present in PCR products"

- In the bioinformatics pipeline, to my understanding, reads that were used for gap-filling had no information about from which sample they were derived; Gaps were filled by using de Bruijn graphs which were constructed based on pooled reads (among all samples). If many conspecific specimens are sequenced simultaneously and they harbor SNPs in these gap regions, what happens? I am afraid that the most abundantly sequenced nucleotide type may "overwrite" sequences for the other conspecifics. Please talk on this concern.
Response: Thanks for pointing this out. Yes, according to the algorithm of de Bruijn graphs, single nucleotide variants (SNVs), if locate in the gap filling region, which is, in our COI case, around 200 bp long, can potentially be overwritten by the most abundant genotypes. But correct assembly can be achieved by using large Kmers (our current algorithm applies Kmer >= 100) in the case of haplotypes. In fact, samples A11 and A12 in plate2 showed a proper example: where A11 and A12 are the same species but possess 2 SNVs in the middle part (286G, 298T for A11;286A,298T for A12), and our pipeline was able to assemble both into correct barcodes.
286 298 300
A12_Hiseq GCCTCCTTCTTTGACCCTACTTTTAGCTAGTAG
A12_sanger GCCTCCTTCTTTGACCCTACTTTTAGCTAGTAG
A11_Hiseq ACCTCCTTCTTTGACCCTGCTTTTAGCTAGAAG
A11_sanger ACCTCCTTCTTTGACCCTGCTTTTAGCTAGAAG

Despite the success, we acknowledge the concerns about potential mis-assemblies, especially for subtle variations in the gap region. Therefore, we have modified our pipeline to also provide an additional sequence to accommodate possible alternative haplotypes and have the users notified by the program.

- "de brujin" is typo (page 7).
Response: Corrected.

Reviewer #2: General comments

In this study, the authors proposed an extremely efficient method for sequencing barcode DNA of identified specimens and for fattening out reference barcode sequence database.
This method may be very important and useful for barcoding, metabarcoding and mitometagenome skimming studies because the reference sequence database is crucial for bridging nucleotide sequences and taxonomic names and because taxonomic names are required for applying existing biological knowledges to barcoding, metabarcoding and mitometagenome skimming studies.
Thus, I strongly recommend to publish this study at Gigascience with several corrections of minor problems listed below.

The largest problem in this study is redistribution of USEARCH which is closed-source non-free software and redistribution is not allowed but included in the distributed file.
Therefore, I recommend to replace USEARCH to VSEARCH which is free and open-source alternative of USEARCH or just exclude USEARCH from distribution.

Response: Many thanks for your kindly suggestion. We have replaced “USEARCH” with

“VSEARCH” in our pipeline, and confirmed that VSEARCH provided identical results. The sentence “sequences were clustered using VSEARCH” was added in line 18 page 7.

The secondary problem is possibility of misassembly of very similar sequences.
If misassembled sequences are registered to the reference sequence database, such sequences might cause misidentification of query sequences.
In order to avoid such possibilities, misassembled or misidentified sequences should be excluded from reference sequence database.
The proposed method assemble short-read Illumina sequences based on k-mer sequence matches and such misassembly was not observed in their real data, but it’s still possible theoretically.
Thus, I recommend to add a function to warn users of a possibility of misassembly if same or similar scored assembly paths exist.
Such warning function can help users to detect problematic sequences.

Response: Many thanks again and we have modified our program so that it produces additional sequences which have similar or same scores comparing to their best alternative. The pipeline also provides an additional note file with a suffix of “note.txt” with notes alerting users about the possibilities. At last, we have also added explanation in the manual of our program.

Specific comments

P4L42 Add "of" to behind of "accuracy".
Response: Added.

P7L42 The authors wrote "much more sensitive" but did not write "than what?".
Response: We have modified this sentence to “the new pipeline is much more sensitive than Sanger in recovering amplicons at low quantity.”

P7L60 Material -> Materials.
Response: Corrected.

P8L35 3uL of 10x reaction buffer was added but total reaction mixture was 25uL. Why?
Response: We do not completely understand the question. Perhaps the phrase “10x” is confusing. It refers to concentration of the buffer rather than volume. Therefore, in this case, the volume of the reaction buffer is 3µl (of 10X concentration), and the total reaction mixture was 25µl.

P8L40 I think this is not a "touchdown" PCR because the annealing temperature of first several cycles is lower than that of the following cycles.
Response: “touchdown” has been removed.

P8L60 Add "also" between "was" and "sequenced".
Response: Added.

P21L45 Add "illustration" between "Schematic" and "of".
Response: Added.
