# Supplementary material for: Filling reference gaps via assembling DNA barcodes using high-throughput sequencing—moving toward barcoding the world
Source: Gigascience. 2017 Oct 25;6(12):1–8. doi: 10.1093/gigascience/gix104 (PMC5726475; doi:10.1093/gigascience/gix104)
Supplement: gix104_Response-to-Reviewer-Comments_Revision-1.pdf [file gix104_response-to-reviewer-comments_revision-1.pdf]

Author's Response To Reviewer Comments

Dear Scott,

We have added the protocol into protocols.io and integrated the DOI (dx.doi.org/10.17504/protocols.io.ka9csh6) in the revised article. We also have checked and completed the spreadsheet from GigaDB to complete the related GigaDB dataset page. Thank you for all your help and looking forward to hearing from you soon.

Sincerely yours,
Xin Zhou
