# Supplementary material for: Filling reference gaps via assembling DNA barcodes using high-throughput sequencing—moving toward barcoding the world
Source: Gigascience. 2017 Oct 25;6(12):1–8. doi: 10.1093/gigascience/gix104 (PMC5726475; doi:10.1093/gigascience/gix104)
Supplement: gix104_Reviewer-1-Report-(Original-Submission).pdf [file gix104_reviewer-1-report-(original-submission).pdf]

Reviewer Report

Title: Filling reference gaps via assembling DNA barcodes using high-throughput sequencing - moving toward barcoding the world

Version: Original Submission **Date:** 8/29/2017

Reviewer name: Seikoh Saitoh

Reviewer Comments to Author:

The manuscript describes a novel method to recover full COI barcodes for individual specimens using high throughput sequencing technology for construction of reference databases linking species names and the barcode sequences, which are crucial for assessments of biodiversity based on molecular methods like metabarcoding.
The method overcomes a problem of Illumina platform (i.e. length of reads [typically150+150 bp] is short for standard COI barcode [>600 bp]). Compared with previously published method for the same purpose (ref.21 Shokralla et al. 2015), the method was improved in cost effectiveness, thanks to the simplified laboratory protocol and superb bioinformatics procedures, although one of the basic ideas (gap-filling) were already published (ref. 23). Further, the authors validated their method by using Pacbio, another (expensive) sequencing platform.
The paper will contribute to studies on biodiversity and meets the scope of the journal. I recommend it be accepted for publication after minor revision.

- Please clarify in the figure legend that sequence logos shown in Fig 4 were based on alignments of Illumina raw reads onto assembled HIFI-barcodes.
- What does "heterozygote" mean for mitochondrial genes? Do you mean heteroplasmy?
- I would suggest the authors talk potential co-amplification of nuclear mitochondrial pseudogenes (so-called numts) by PCR.
- In the bioinformatics pipeline, to my understanding, reads that were used for gap-filling had no information about from which sample they were derived; Gaps were filled by using de Bruijn graphs which were constructed based on pooled reads (among all samples). If many conspecific specimens are sequenced simultaneously and they harbor SNPs in these gap regions, what happens? I am afraid that the most abundantly sequenced nucleotide type may "overwrite" sequences for the other conspecifics. Please talk on this concern.
- "de brujin" is typo (page 7).

Methods

Are the methods appropriate to the aims of the study, are they well described, and are necessary controls included? Yes

Conclusions

Are the conclusions adequately supported by the data shown? Yes

Reporting Standards

Does the manuscript adhere to the journal's guidelines on [minimum standards of reporting?]() Yes Choose an item.

Statistics

Are you able to assess all statistics in the manuscript, including the appropriateness of statistical tests used? Yes, and I have assessed the statistics in my report.

Quality of Written English

Please indicate the quality of language in the manuscript: Acceptable

Declaration of Competing Interests

Please complete a declaration of competing interests, considering the following questions:

- Have you in the past five years received reimbursements, fees, funding, or salary from an organisation that may in any way gain or lose financially from the publication of this manuscript, either now or in the future?
- Do you hold any stocks or shares in an organisation that may in any way gain or lose financially from the publication of this manuscript, either now or in the future?
- Do you hold or are you currently applying for any patents relating to the content of the manuscript?
- Have you received reimbursements, fees, funding, or salary from an organization that holds or has applied for patents relating to the content of the manuscript?
- Do you have any other financial competing interests?
- Do you have any non-financial competing interests in relation to this paper?

If you can answer no to all of the above, write 'I declare that I have no competing interests' below. If your reply is yes to any, please give details below.

I declare that I have no competing interests.

I agree to the open peer review policy of the journal. I understand that my name will be included on my report to the authors and, if the manuscript is accepted for publication, my named report including any attachments I upload will be posted on the website along with the authors' responses. I agree for my report to be made available under an Open Access Creative Commons CC-BY license (http://creativecommons.org/licenses/by/4.0/). I understand that any comments which I do not wish to be included in my named report can be included as confidential comments to the editors, which will not be published.

I agree to the open peer review policy of the journal

To further support our reviewers, we have joined with Publons, where you can gain additional credit to further highlight your hard work (see: https://publons.com/journal/530/gigascience). On publication of this paper, your review will be automatically added to Publons, you can then choose whether or not to claim your Publons credit. I understand this statement.

Yes
