# Supplementary material for: Filling reference gaps via assembling DNA barcodes using high-throughput sequencing—moving toward barcoding the world
Source: Gigascience. 2017 Oct 25;6(12):1–8. doi: 10.1093/gigascience/gix104 (PMC5726475; doi:10.1093/gigascience/gix104)
Supplement: gix104_Reviewer-2-Report-(Original-Submission).pdf [file gix104_reviewer-2-report-(original-submission).pdf]

Reviewer Report

Title: Filling reference gaps via assembling DNA barcodes using high-throughput sequencing - moving toward barcoding the world

Version: Original Submission **Date:** 9/24/2017

Reviewer name: Akifumi Tanabe

Reviewer Comments to Author:

General comments

In this study, the authors proposed an extremely efficient method for sequencing barcode DNA of identified specimens and for fattening out reference barcode sequence database.
This method may be very important and useful for barcoding, metabarcoding and mitometagenome skimming studies because the reference sequence database is crucial for bridging nucleotide sequences and taxonomic names and because taxonomic names are required for applying existing biological knowledges to barcoding, metabarcoding and mitometagenome skimming studies.
Thus, I strongly recommend to publish this study at Gigascience with several corrections of minor problems listed below.

The largest problem in this study is redistribution of USEARCH which is closed-source non-free software and redistribution is not allowed but included in the distributed file.
Therefore, I recommend to replace USEARCH to VSEARCH which is free and open-source alternative of USEARCH or just exclude USEARCH from distribution.

The secondary problem is possibility of misassembly of very similar sequences.
If misassembled sequences are registered to the reference sequence database, such sequences might cause misidentification of query sequences.
In order to avoid such possibilities, misassembled or misidentified sequences should be excluded from reference sequence database.
The proposed method assemble short-read Illumina sequences based on k-mer sequence matches and such misassembly was not observed in their real data, but its still possible theoretically.
Thus, I recommend to add a function to warn users of a possibility of misassembly if same or similar scored assembly paths exist.
Such warning function can help users to detect problematic sequences.

Specific comments

P4L42 Add "of" to behind of "accuracy".
P7L42 The authors wrote "much more sensitive" but did not write "than what?".

P7L60 Material -> Materials.
P8L35 3uL of 10x reaction buffer was added but total reaction mixture was 25uL. Why?
P8L40 I think this is not a "touchdown" PCR because the annealing temperature of first several cycles is lower than that of the following cycles.
P8L60 Add "also" between "was" and "sequenced".
P21L45 Add "illustration" between "Schematic" and "of".

Methods

Are the methods appropriate to the aims of the study, are they well described, and are necessary controls included? Yes

Conclusions

Are the conclusions adequately supported by the data shown? Yes

Reporting Standards

Does the manuscript adhere to the journal's guidelines on minimum standards of reporting? Yes Choose an item.

Statistics

Are you able to assess all statistics in the manuscript, including the appropriateness of statistical tests used? Yes, and I have assessed the statistics in my report.

Quality of Written English

Please indicate the quality of language in the manuscript: Acceptable

Declaration of Competing Interests

Please complete a declaration of competing interests, considering the following questions:

- Have you in the past five years received reimbursements, fees, funding, or salary from an organisation that may in any way gain or lose financially from the publication of this manuscript, either now or in the future?
- Do you hold any stocks or shares in an organisation that may in any way gain or lose financially from the publication of this manuscript, either now or in the future?
- Do you hold or are you currently applying for any patents relating to the content of the manuscript?
- Have you received reimbursements, fees, funding, or salary from an organization that holds or has applied for patents relating to the content of the manuscript?
- Do you have any other financial competing interests?

- Do you have any non-financial competing interests in relation to this paper?

If you can answer no to all of the above, write 'I declare that I have no competing interests' below. If your reply is yes to any, please give details below.

I declare that I have no competing interests.

I agree to the open peer review policy of the journal. I understand that my name will be included on my report to the authors and, if the manuscript is accepted for publication, my named report including any attachments I upload will be posted on the website along with the authors' responses. I agree for my report to be made available under an Open Access Creative Commons CC-BY license (http://creativecommons.org/licenses/by/4.0/). I understand that any comments which I do not wish to be included in my named report can be included as confidential comments to the editors, which will not be published.

I agree to the open peer review policy of the journal

To further support our reviewers, we have joined with Publons, where you can gain additional credit to further highlight your hard work (see: https://publons.com/journal/530/gigascience). On publication of this paper, your review will be automatically added to Publons, you can then choose whether or not to claim your Publons credit. I understand this statement.

Yes
